# Global, regional and national burden of traumatic amputations from 1990 to 2021: a systematic analysis of the Global Burden of Disease study 2021

**DOI:** 10.3389/fpubh.2025.1583523

**Published:** 2025-06-02

**Authors:** Baixing Wei, Jie Zhang, Yuanpei Cheng, Han Wu

**Affiliations:** Department of Orthopedics, China-Japan Union Hospital of Jilin University, Changchun, China

**Keywords:** global health, disease burden, epidemiology, trauma, amputation

## Abstract

**Background:**

Traumatic amputations are serious public health problems with enormous health and economic costs, but to date there has been little research on the epidemiology of traumatic amputations. We aimed to comprehensively analyze the global burden of disease due to traumatic amputations during 1990–2021 and to project trends over the next 15 years.

**Method:**

Global Burden of Disease (GBD) 2021 data were used to demonstrate the burden of traumatic amputations in different populations, including incidence, prevalence, and years lived with disability (YLDs). In-depth analyses and projections were performed using Age-Period-Cohort (APC) model analysis, decomposition analysis, and Autoregressive Integrated Moving Average (ARIMA) models.

**Result:**

There was an increase in the number of traumatic amputations globally in 2021 compared to 1990. However, the change in age-standardized rates from 1990 to 2021 declined significantly. The association between the Socio-demographic Index (SDI) and the burden of amputations in 2021 was not linear. Results of the APC analysis indicated that the burden was lower in the later-born cohort and showed an overall decreasing trend over time. The results of the decomposition analyses indicated that in most cases age change suppressed incidence but promoted increases in prevalence and YLDs. Population changes increased the burden of amputations, while epidemiologic changes did the opposite. The burden was projected to trend downward globally and in most regions over the next 15 years. Finally, mechanical forces and falls were the two most prominent factors.

**Conclusion:**

The global burden of traumatic amputations increased in number from 1990 to 2021, but the age-standardized rate declined significantly and was expected to continue to decline in the future. Population growth is now the main cause of the burden, and more attention needs to be paid to men, youth and the older adult.

## 1 Introduction

Amputation is used as a medical treatment in the treatment of severe injuries that are life-threatening or in which the function of the limbs cannot be restored, and results in the loss of a part of the body, such as upper limbs, lower limbs, fingers or toes. Regardless of the type of amputation, it implies a serious loss of mobility and loss of personal image, which brings heavy physical and psychological harm to the patient. Amputation also imposes a huge economic burden on families and society, and is a global public health problem that needs to be addressed urgently (1–5). Common causes of amputation can be categorized into non-traumatic diseases such as diabetes or peripheral vascular disease (6), and traumatic injuries due to a number of causes (1). The etiologic structure of amputations is related to the local level of economic development. For example, trauma is the leading cause of leg amputations in developing countries, whereas in developed countries the most common cause is peripheral vascular disease (1). Rapid economic development will bring more attention to non-traumatic amputations, however, for traumatic ones, the characteristics of younger age and longer life expectancy of the patients dictate that analyzing the epidemiological changes in traumatic amputations is still important (1). However, not much research has been done (7, 8).

The Global Burden of Disease (GBD) database is a database that quantifies the extent to which health issues such as diseases, injuries, and risk factors affect the health of populations at the global, regional, and national levels (9). In conjunction with the above, in this study we focused on all types of traumatic amputations and used the GBD database to obtain comprehensive data, visualize and describe them, and then analyzed them using advanced methods such as age-period-cohort (APC) and decomposition analyses. Future burden was also projected using ARIMA. An attempt was made to update the global understanding of epidemiologic trends in traumatic amputations.

In summary, the aim of our work is to provide a rationale for scrutinizing existing health policies and to motivate policymakers, healthcare providers, and researchers to propose prospective, targeted interventions that will reduce the burden of traumatic amputation and improve the prognosis for patients.

## 2 Method

### 2.1 Data sources

The GBD 2021 database provides comprehensive epidemiologic data on 371 diseases and injuries for 204 countries and territories from 1990 to 2021 (9). The resource search, data processing steps and modeling methodology for the GBD database have been described in detail (9). We extracted incidence, prevalence, and years lived with disability (YLDs) data from the GBD results tool ^1^for 1990–2021 for different countries and regions, gender, and age of traumatic amputation populations. Traumatic amputations here included all categories given within this tool, including unilateral or bilateral amputations of the upper or lower limbs, fingers (except thumbs), thumbs, and toes. 7 total categories.

### 2.2 Conceptual explanations

YLD is the sum of years of survival to health loss calculated by using the estimated age-sex-location-year-specific (in this study, traumatic amputation) disability weighting of sequelae. Using the GBD standard population structure, we present estimates in terms of number and age- standardized rates per 100,000 population. Socio-demographic Index (SDI) is a quantitative assessment of a society’s level of development based on educational attainment, fertility and income per capita and assigned a value of 0–1, based on which countries are categorized into five SDI regions ranging from low to high. Age standardization is the process of adjusting rates for different population groups to eliminate the interference of differences in age composition, thus providing a fair comparison of relevant rates between groups of people (9).

### 2.3 Data organization and visualization

The data obtained from the GBD database were organized to present the prevalence, incidence, and YLDs of the population by time, region, gender and age, and cause of injury in multiple tables, and were plotted in R to visualize the data for the above subgroups.

The world map was created using the map_data (‘world’) function of ggplot2 in the R package and colored according to the acquired age-standardized data in numerical segments to show the geographic distribution of the burden of amputation for trauma etiology in different countries and regions around the world.

### 2.4 Age-period-cohort model

Age-period-cohort (APC) model (10, 11) is a tool used in epidemiology to explain and analyze the influence of age effects, period effects, and cohort effects on specific outcomes or trends. Age effects refer to differences in the burden of disease for amputations in different age groups caused by increasing age. Period effects refer to differences in the burden of disease for amputations due to factors acting simultaneously for all age groups at a given point in time. Cohort effects refer to changes in the burden of disease for amputation due to different risk factors for different birth years.

Dividing age and year into consecutive 5-year intervals (1992–2021 was used for year here because 1990–1991 did not span a 5-year interval), in addition, we summarized 25 birth cohorts from 1893–1901 to 2013–2021 based on the law of birth cohort = period-age. Age, period, and cohort average were chosen as the reference group. The relative risk (RR) values for each age, period, and cohort represent the independent risk compared with the reference group.

### 2.5 Decomposition analysis

Decomposition analysis (12, 13) is a method of delineating different factors by means of mathematical decomposition and identifying how they affect overall change. To understand the factors driving changes in the burden of traumatic amputations between 1990 and 2021, we used Das Gupta’s decomposition analysis method (12, 13). The specific contributions of the three factors driving changes in the burden of disease (i.e., age, epidemiological trends, and population) were visualized in R. The black dots represented the values resulting from the combined effects of the three factors.

### 2.6 ARIMA prediction model

Autoregressive Integrated Moving Average (14) (ARIMA) is a statistical model widely used in time series analysis and forecasting, and is a differential integrated moving average autoregressive model. ARIMA consists of an autoregressive (AR) model, a difference (I) and a moving average (MA) model. ARIMA is denoted by (p, d, q), p is the autoregressive order. d is the order of difference. q is the moving average order.

In this study, the ARIMA model was used to analyze the trend of disease incidence of amputation and to predict the burden of disease for the next 15 years globally and in different SDI regions. Auto.arima() function was used to select the best optimized model (14). The normality of model residuals was tested by observing QQ plots, ACF and PACF plots. The residual series were tested for white noise using Ljung-Box.

### 2.7 Statistical analysis

All statistical analyses and data visualizations were performed using R (version 4.3.2). Statistical results are presented as means and 95% uncertainty intervals (UI) or 95% confidence intervals (CI). For trend analysis, *p* < 0.05 was considered statistically significant.

### 2.8 Patient and public involvement statement

The study used a publicly available database with no patient participation.

## 3 Results

### 3.1 Overview of the Global Burden of Disease from traumatic amputations

In 2021, there were 445.2 million (95% UI 409.0–485.8) individuals with traumatic amputation globally, 10 million (9.1–12.9) individuals with new cases in 2021 alone, and a YLD for traumatic amputation of 5.9 million (4.0–9.1) years (Table 1). Comparisons with 1990 data are all elevated, with the number of prevalence and YLDs being particularly significant. However, the global age-standardized rate (per 100,000) of incidence, prevalence, and YLDs, declined significantly from 1990 to 2021, to −0.26 (−0.28 to −0.24) per 100,000, −0.24 (−0.26 to −0.22) per 100,000, and −0.32 (−0.37 to −0.28) per 100,000, respectively.

**Table 1:** Number of incidence, prevalence, and YLDs of traumatic amputations at all ages in 2021, and changes in age-standardized rates per 100,000 in countries and areas, 1990 to 2021.

				Incidence			Prevalence			YLDs		
				1990 (thousands)	2021 (thousands)	Change in age-standardized rate (per 100000)	1990 (thousands)	2021 (thousands)	Change in age-standardized rate (per 100000)	1990 (thousands)	2021 (thousands)	Change in age-standardized rate (per 100000)
Global				10352 (8657, 12308)	10859 (9064, 12882)	−0.26 (−0.28, −0.24)	338099 (310645, 370807)	445238 (408968, 485841)	−0.24 (−0.26, −0.22)	4988 (3482, 7332)	5936 (3988, 9067)	−0.32 (−0.37, −0.28)
	Southeast Asia, East Asia, and Oceania			2050 (1740, 2443)	2239 (1838, 2694)	−0.11 (−0.15, −0.07)	66841 (60831, 74500)	99903 (91106, 110708)	−0.11 (−0.13, −0.08)	1238 (898, 1740)	1319 (855, 2070)	−0.39 (−0.49, −0.28)
		East Asia		1242 (1013, 1512)	1425 (1163, 1751)	−0.03 (−0.09, 0.03)	44870 (40642, 50338)	67249 (60890, 74989)	−0.08 (−0.1, −0.04)	851 (608, 1168)	820 (499, 1321)	−0.44 (−0.57, −0.3)
			China	1205 (982, 1467)	1393 (1135, 1716)	−0.02 (−0.08, 0.04)	43610 (39485, 48981)	65819 (59533, 73489)	−0.07 (−0.1, −0.03)	829 (593, 1137)	798 (485, 1289)	−0.44 (−0.57, −0.3)
			Democratic People's Republic of Korea	15 (13, 18)	18 (15, 21)	−0.1 (−0.15, −0.04)	534 (499, 583)	722 (679, 772)	−0.15 (−0.2, −0.12)	11 (8, 15)	13 (9, 19)	−0.28 (−0.35, −0.22)
			Taiwan (Province of China)	21 (18, 26)	14 (12, 17)	−0.42 (−0.46, −0.39)	726 (664, 804)	708 (658, 770)	−0.41 (−0.44, −0.38)	11 (7, 16)	9 (5, 14)	−0.54 (−0.62, −0.48)
		Southeast Asia		802(675,962)	799 (673, 941)	−0.3 (−0.35, −0.26)	21810 (19545, 26224)	32247 (29274, 36388)	−0.22 (−0.24, −0.19)	384 (263, 587)	492 (344, 741)	−0.32 (−0.39, −0.27)
			Cambodia	18 (15, 22)	19 (15, 23)	−0.35 (−0.45, −0.24)	806 (495, 1513)	1034 (778, 1596)	−0.29 (−0.38, −0.19)	25 (10, 62)	23 (12, 45)	−0.48 (−0.56, −0.32)
			Indonesia	249 (204, 300)	254 (212, 302)	−0.28 (−0.31, −0.26)	8088 (7280, 9474)	10773 (9831, 12038)	−0.29 (−0.3, −0.27)	140 (98, 207)	166 (116, 247)	−0.37 (−0.41, −0.32)
			Lao People's Democratic Republic	9 (7, 14)	7 (6, 9)	−0.54 (−0.69, −0.39)	154 (143, 165)	239 (223, 259)	−0.28 (−0.3, −0.26)	3 (2, 4)	4 (3, 6)	−0.38 (−0.43, −0.33)
			Malaysia	20 (17, 25)	32 (27, 38)	−0.14 (−0.18, −0.1)	584 (541, 632)	1155 (1071, 1252)	−0.15 (−0.17, −0.13)	9 (6, 13)	15 (10, 23)	−0.29 (−0.36, −0.22)
			Maldives	0 (0, 0)	1 (0, 1)	−0.25 (−0.31, −0.19)	8 (8, 9)	23 (21, 26)	−0.24 (−0.27, −0.2)	0 (0, 0)	0 (0, 0)	−0.42 (−0.51, −0.33)
			Mauritius	1 (1, 1)	1 (1, 1)	−0.17 (−0.21, −0.13)	38 (35, 41)	50 (46, 54)	−0.16 (−0.19, −0.14)	1 (0, 1)	1 (0, 1)	−0.25 (−0.32, −0.2)
			Myanmar	76 (64, 91)	134 (106, 175)	0.3 (0.07, 0.66)	2371 (1988, 3345)	3411 (2952, 4309)	−0.12 (−0.19, −0.03)	46 (30, 80)	60 (40, 90)	−0.2 (−0.33, −0.07)
			Philippines	116 (97, 141)	112 (95, 131)	−0.44 (−0.49, −0.4)	2803 (2408, 3590)	4413 (3883, 5233)	−0.25 (−0.28, −0.22)	52 (35, 80)	77 (53, 114)	−0.29 (−0.33, −0.25)
			Seychelles	0 (0, 0)	0 (0, 0)	−0.3 (−0.35, −0.25)	3 (3, 4)	4 (4, 5)	−0.26 (−0.29, −0.24)	0 (0, 0)	0 (0, 0)	−0.34 (−0.41, −0.29)
			Sri Lanka	108 (74, 155)	29 (24, 35)	−0.77 (−0.85, −0.67)	1144 (993, 1381)	2261 (1809, 2917)	0.26 (0.09, 0.45)	20 (14, 30)	34 (21, 54)	0.11 (−0.07, 0.28)
			Thailand	94 (78, 114)	75 (64, 90)	−0.26 (−0.31, −0.2)	2925 (2734, 3145)	3870 (3553, 4208)	−0.23 (−0.26, −0.2)	45 (32, 65)	48 (31, 76)	−0.41 (−0.5, −0.33)
			Timor-Leste	4 (3, 6)	1 (1, 2)	−0.77 (−0.85, −0.66)	75 (55, 105)	105 (78, 146)	−0.04 (−0.09, 0.01)	2 (1, 4)	3 (2, 4)	−0.13 (−0.18, −0.07)
			Viet Nam	104 (84, 129)	133 (108, 159)	−0.14 (−0.21, −0.08)	2779 (2565, 3006)	4862 (4508, 5261)	−0.14 (−0.16, −0.1)	41 (29, 60)	62 (40, 95)	−0.27 (−0.35, −0.21)
		Oceania		6 (5, 7)	14 (12, 17)	0.07 (0.01, 0.14)	160 (148, 175)	408 (369, 455)	0.07 (0.04, 0.12)	3 (2, 4)	8 (6, 11)	0.09 (0.03, 0.14)
			American Samoa	0 (0, 0)	0 (0, 0)	−0.21 (−0.25, −0.16)	2 (1, 2)	2 (2, 2)	−0.1 (−0.14, −0.04)	0 (0, 0)	0 (0, 0)	−0.08 (−0.14, 0)
			Cook Islands	0 (0, 0)	0 (0, 0)	−0.28 (−0.4, −0.17)	1 (0, 1)	1 (1, 1)	−0.02 (−0.06, 0.03)	0 (0, 0)	0 (0, 0)	−0.27 (−0.38, −0.16)
			Fiji	1 (1, 1)	1 (1, 1)	−0.13 (−0.17, −0.08)	19 (18, 21)	25 (23, 27)	−0.14 (−0.16, −0.11)	0 (0, 0)	0 (0, 1)	−0.16 (−0.21, −0.11)
			Guam	0 (0, 0)	0 (0, 0)	−0.22 (−0.26, −0.16)	4 (4, 5)	5 (5, 6)	−0.18 (−0.2, −0.15)	0 (0, 0)	0 (0, 0)	−0.17 (−0.22, −0.13)
			Kiribati	0 (0, 0)	0 (0, 0)	−0.22 (−0.26, −0.18)	2 (1, 2)	2 (2, 3)	−0.17 (−0.2, −0.14)	0 (0, 0)	0 (0, 0)	−0.23 (−0.29, −0.17)
			Marshall Islands	0 (0, 0)	0 (0, 0)	−0.15 (−0.2, −0.11)	1 (1, 1)	2 (1, 2)	−0.15 (−0.17, −0.13)	0 (0, 0)	0 (0, 0)	−0.19 (−0.23, −0.15)
			Micronesia (Federated States of)	0 (0, 0)	0 (0, 0)	−0.11 (−0.15, −0.06)	3 (3, 3)	3 (3, 4)	−0.08 (−0.11, −0.05)	0 (0, 0)	0 (0, 0)	−0.16 (−0.22, −0.1)
			Nauru	0 (0, 0)	0 (0, 0)	−0.08 (−0.13, −0.02)	0 (0, 0)	0 (0, 0)	−0.11 (−0.14, −0.09)	0 (0, 0)	0 (0, 0)	−0.15 (−0.19, −0.11)
			Niue	0 (0, 0)	0 (0, 0)	−0.15 (−0.2, −0.1)	0 (0, 0)	0 (0, 0)	−0.1 (−0.12, −0.06)	0 (0, 0)	0 (0, 0)	−0.2 (−0.26, −0.14)
			Northern Mariana Islands	0 (0, 0)	0 (0, 0)	−0.24 (−0.29, −0.17)	2 (2, 2)	2 (2, 2)	−0.23 (−0.26, −0.2)	0 (0, 0)	0 (0, 0)	−0.24 (−0.28, −0.2)
			Palau	0 (0, 0)	0 (0, 0)	−0.03 (−0.08, 0.04)	1 (1, 1)	1 (1, 1)	−0.03 (−0.05, 0)	0 (0, 0)	0 (0, 0)	−0.13 (−0.19, −0.07)
			Papua New Guinea	4 (3, 5)	11 (9, 14)	0.14 (0.07, 0.21)	96 (88, 106)	306 (276, 344)	0.15 (0.11, 0.21)	2 (1, 3)	6 (4, 8)	0.15 (0.09, 0.23)
			Samoa	0 (0, 0)	0 (0, 0)	−0.26 (−0.35, −0.18)	4 (4, 5)	6 (6, 7)	0 (−0.05, 0.08)	0 (0, 0)	0 (0, 0)	−0.06 (−0.14, 0.04)
			Solomon Islands	0 (0, 0)	1 (1, 1)	0.01 (−0.06, 0.08)	9 (9, 10)	23 (20, 25)	−0.01 (−0.04, 0.03)	0 (0, 0)	0 (0, 1)	−0.08 (−0.14, −0.02)
			Tokelau	0 (0, 0)	0 (0, 0)	−0.16 (−0.21, −0.1)	0 (0, 0)	0 (0, 0)	−0.16 (−0.18, −0.13)	0 (0, 0)	0 (0, 0)	−0.29 (−0.36, −0.22)
			Tonga	0 (0, 0)	0 (0, 0)	−0.25 (−0.3, −0.2)	2 (2, 2)	2 (2, 2)	−0.19 (−0.22, −0.17)	0 (0, 0)	0 (0, 0)	−0.27 (−0.33, −0.22)
			Tuvalu	0 (0, 0)	0 (0, 0)	−0.12 (−0.16, −0.07)	0 (0, 0)	0 (0, 0)	−0.13 (−0.15, −0.1)	0 (0, 0)	0 (0, 0)	−0.24 (−0.3, −0.19)
			Vanuatu	0 (0, 0)	0 (0, 0)	−0.13 (−0.17, −0.09)	4 (3, 4)	8 (7, 9)	−0.12 (−0.14, −0.1)	0 (0, 0)	0 (0, 0)	−0.12 (−0.17, −0.08)
	Central Europe, Eastern Europe and Central Asia			2064(1646,2544)	1411(1118,1733)	−0.29 (−0.31, −0.28)	80128 (73869, 87202)	66548 (61429, 72593)	−0.28 (−0.29, −0.27)	954 (622, 1487)	681 (407, 1148)	−0.38 (−0.45, −0.33)
		Central Asia		219 (176, 273)	233 (187, 290)	−0.2 (−0.22, −0.18)	6799 (6249, 7428)	8818 (8154, 9666)	−0.19 (−0.21, −0.17)	88 (60, 132)	105 (69, 165)	−0.26 (−0.3, −0.21)
			Armenia	14 (11, 17)	7 (5, 8)	−0.39 (−0.43, −0.36)	525 (482, 581)	359 (328, 397)	−0.35 (−0.39, −0.31)	7 (4, 10)	4 (3, 7)	−0.41 (−0.49, −0.35)
			Azerbaijan	20 (16, 25)	23 (18, 29)	−0.13 (−0.17, −0.09)	633 (580, 696)	1029 (938, 1136)	−0.07 (−0.11, −0.02)	8 (6, 13)	12 (8, 19)	−0.19 (−0.28, −0.11)
			Georgia	19 (15, 24)	12 (10, 15)	0.09 (0.02, 0.17)	760 (696, 833)	599 (553, 656)	0.07 (0.03, 0.11)	9 (6, 14)	7 (4, 11)	0.02 (−0.05, 0.06)
			Kazakhstan	64 (51, 81)	59 (47, 75)	−0.18 (−0.23, −0.14)	2047 (1881, 2234)	2092 (1928, 2298)	−0.2 (−0.22, −0.17)	26 (18, 40)	23 (14, 38)	−0.32 (−0.39, −0.26)
			Kyrgyzstan	13 (11, 16)	15 (12, 18)	−0.26 (−0.3, −0.22)	393 (358, 436)	477 (441, 524)	−0.28 (−0.31, −0.26)	5 (4, 8)	6 (4, 9)	−0.39 (−0.45, −0.33)
			Mongolia	8 (6, 9)	11 (9, 14)	0 (−0.05, 0.06)	190 (175, 209)	371 (340, 408)	−0.01 (−0.04, 0.02)	3 (2, 4)	5 (3, 7)	−0.18 (−0.27, −0.11)
			Tajikistan	14 (12, 18)	20 (16, 25)	−0.23 (−0.26, −0.2)	381 (345, 422)	779 (691, 881)	−0.06 (−0.13, 0.04)	5 (4, 8)	12 (8, 18)	0.05 (−0.08, 0.24)
			Turkmenistan	10 (8, 13)	13 (10, 17)	0 (−0.07, 0.08)	284 (257, 317)	464 (426, 513)	−0.04 (−0.08, 0)	4 (3, 6)	5 (3, 8)	−0.18 (−0.25, −0.1)
			Uzbekistan	57 (45, 70)	73 (59, 88)	−0.16 (−0.19, −0.12)	1585 (1444, 1749)	2649 (2447, 2884)	−0.17 (−0.2, −0.15)	20 (14, 31)	32 (21, 50)	−0.23 (−0.28, −0.19)
		Central Europe		733(569,925)	475 (363, 597)	−0.25 (−0.27, −0.23)	28662 (26343, 31356)	24196 (22312, 26546)	−0.23 (−0.24, −0.22)	324 (209, 515)	222 (120, 395)	−0.38 (−0.49, −0.3)
			Albania	27 (21, 36)	15 (11, 19)	−0.26 (−0.31, −0.2)	764 (690, 844)	677 (621, 745)	−0.23 (−0.26, −0.2)	8 (5, 13)	6 (3, 11)	−0.37 (−0.48, −0.29)
			Bosnia and Herzegovina	37 (29, 49)	13 (10, 16)	−0.47 (−0.53, −0.41)	1168 (1058, 1296)	785 (708, 875)	−0.26 (−0.3, −0.21)	13 (8, 21)	8 (5, 14)	−0.3 (−0.39, −0.23)
			Bulgaria	49 (38, 61)	32 (24, 42)	−0.11 (−0.17, −0.06)	2245 (2064, 2450)	1712 (1571, 1879)	−0.15 (−0.17, −0.12)	25 (16, 40)	17 (10, 29)	−0.26 (−0.33, −0.2)
			Croatia	27 (21, 34)	16 (13, 20)	−0.29 (−0.34, −0.23)	1121 (1026, 1229)	845 (773, 927)	−0.25 (−0.28, −0.21)	11 (6, 19)	8 (4, 14)	−0.29 (−0.36, −0.25)
			Czechia	69 (53, 88)	46 (35, 58)	−0.31 (−0.33, −0.27)	2797 (2566, 3059)	2410 (2210, 2649)	−0.27 (−0.29, −0.26)	29 (18, 48)	21 (11, 38)	−0.39 (−0.48, −0.33)
			Hungary	56 (44, 70)	36 (28, 46)	−0.24 (−0.28, −0.2)	2308 (2115, 2529)	1904 (1742, 2089)	−0.2 (−0.22, −0.18)	27 (17, 42)	17 (9, 31)	−0.39 (−0.51, −0.29)
			North Macedonia	10 (8, 13)	9 (7, 11)	−0.11 (−0.17, −0.05)	377 (346, 414)	444 (405, 490)	−0.13 (−0.16, −0.1)	4 (3, 7)	4 (2, 7)	−0.26 (−0.37, −0.19)
			Montenegro	3 (2, 4)	2 (2, 3)	−0.14 (−0.16, −0.1)	119 (109, 131)	120 (109, 132)	−0.13 (−0.15, −0.12)	1 (1, 2)	1 (1, 2)	−0.21 (−0.28, −0.17)
			Poland	205 (160, 260)	145 (112, 183)	−0.26 (−0.29, −0.23)	7870 (7232, 8618)	7145 (6581, 7829)	−0.25 (−0.27, −0.24)	92 (60, 143)	65 (35, 116)	−0.43 (−0.54, −0.34)
			Romania	142 (110, 183)	82 (62, 104)	−0.24 (−0.28, −0.2)	5679 (5199, 6231)	4161 (3826, 4558)	−0.25 (−0.26, −0.23)	68 (45, 106)	39 (21, 68)	−0.42 (−0.53, −0.33)
			Serbia	47 (36, 60)	35 (26, 44)	−0.15 (−0.19, −0.11)	1974 (1804, 2172)	1837 (1677, 2021)	−0.13 (−0.15, −0.1)	22 (14, 35)	17 (9, 30)	−0.29 (−0.42, −0.2)
			Slovakia	33 (25, 43)	26 (20, 33)	−0.19 (−0.22, −0.15)	1209 (1109, 1332)	1250 (1144, 1378)	−0.17 (−0.19, −0.15)	13 (8, 22)	11 (6, 20)	−0.33 (−0.45, −0.25)
			Slovenia	15 (12, 20)	11 (9, 14)	−0.27 (−0.32, −0.23)	571 (525, 625)	555 (511, 609)	−0.22 (−0.24, −0.2)	6 (3, 10)	5 (3, 9)	−0.3 (−0.37, −0.26)
		Eastern Europe		1112(893,1370)	703 (564, 860)	−0.29 (−0.32, −0.27)	44667 (41090, 48724)	33534 (30874, 36826)	−0.29 (−0.31, −0.27)	542 (354, 842)	354 (214, 588)	−0.39 (−0.46, −0.34)
			Belarus	41 (33, 50)	31 (25, 38)	−0.12 (−0.16, −0.08)	1665 (1526, 1824)	1492 (1362, 1645)	−0.14 (−0.16, −0.12)	20 (13, 31)	14 (8, 25)	−0.31 (−0.43, −0.23)
			Estonia	8 (6, 9)	4 (3, 5)	−0.37 (−0.4, −0.34)	308 (284, 336)	193 (177, 212)	−0.34 (−0.36, −0.33)	4 (2, 6)	2 (1, 3)	−0.5 (−0.59, −0.42)
			Latvia	14 (11, 17)	6 (5, 7)	−0.36 (−0.4, −0.32)	557 (513, 608)	294 (272, 322)	−0.35 (−0.36, −0.34)	7 (5, 11)	3 (2, 5)	−0.48 (−0.56, −0.42)
			Lithuania	17 (13, 20)	8 (7, 10)	−0.29 (−0.32, −0.25)	657 (604, 719)	419 (385, 458)	−0.29 (−0.3, −0.27)	8 (5, 12)	4 (3, 7)	−0.4 (−0.47, −0.34)
			Republic of Moldova	18 (15, 23)	9 (7, 10)	−0.37 (−0.4, −0.34)	693 (637, 755)	454 (419, 495)	−0.35 (−0.37, −0.34)	9 (6, 13)	5 (3, 8)	−0.46 (−0.52, −0.4)
			Russian Federation	805 (643, 992)	499 (400, 612)	−0.35 (−0.38, −0.31)	31598 (29141, 34404)	23201 (21350, 25470)	−0.34 (−0.36, −0.31)	382 (248, 592)	240 (143, 403)	−0.44 (−0.51, −0.38)
			Ukraine	210 (167, 258)	146 (118, 178)	−0.11 (−0.15, −0.07)	9190 (8358, 10147)	7480 (6875, 8225)	−0.14 (−0.17, −0.11)	113 (75, 176)	85 (53, 137)	−0.21 (−0.26, −0.16)
	High-income			1664(1278,2128)	1385 (1062, 1769)	−0.25 (−0.27, −0.23)	74999 (68636, 82493)	77304 (70766, 84896)	−0.24 (−0.25, −0.23)	729 (406, 1279)	731 (396, 1288)	−0.26 (−0.29, −0.25)
		High-income Asia Pacific		343(264,444)	217 (166, 278)	−0.32 (−0.34, −0.29)	14544 (13319, 15966)	13892 (12757, 15213)	−0.28 (−0.29, −0.26)	143 (79, 247)	127 (68, 224)	−0.32 (−0.37, −0.29)
			Brunei Darussalam	1 (0, 1)	1 (1, 1)	−0.16 (−0.2, −0.13)	16 (15, 18)	31 (28, 34)	−0.17 (−0.19, −0.15)	0 (0, 0)	0 (0, 1)	−0.28 (−0.36, −0.22)
			Japan	219 (166, 283)	137 (105, 175)	−0.28 (−0.3, −0.26)	10392 (9544, 11402)	9327 (8578, 10225)	−0.25 (−0.27, −0.24)	96 (51, 169)	85 (45, 149)	−0.26 (−0.28, −0.25)
			Republic of Korea	117 (91, 151)	72 (55, 93)	−0.39 (−0.43, −0.34)	3910 (3562, 4333)	4066 (3720, 4489)	−0.36 (−0.38, −0.35)	45 (28, 73)	38 (20, 67)	−0.48 (−0.57, −0.42)
			Singapore	6 (5, 8)	8 (6, 10)	−0.17 (−0.22, −0.13)	227 (206, 251)	469 (425, 519)	−0.12 (−0.14, −0.11)	2 (1, 4)	4 (2, 8)	−0.21 (−0.3, −0.15)
		Australasia		80 (59, 107)	89 (66, 118)	−0.2 (−0.24, −0.16)	3483 (3116, 3928)	4834 (4327, 5434)	−0.18 (−0.2, −0.16)	30 (15, 56)	42 (21, 78)	−0.17 (−0.19, −0.14)
			Australia	65 (48, 87)	71 (52, 94)	−0.21 (−0.26, −0.17)	2836 (2528, 3206)	3912 (3496, 4402)	−0.19 (−0.21, −0.16)	24 (12, 46)	34 (17, 63)	−0.18 (−0.2, −0.15)
			New Zealand	15 (11, 20)	18 (13, 24)	−0.15 (−0.2, −0.09)	647 (576, 728)	923 (829, 1039)	−0.15 (−0.18, −0.11)	6 (3, 10)	8 (4, 15)	−0.15 (−0.19, −0.11)
		Western Europe		641(495,818)	514 (395, 664)	−0.22 (−0.25, −0.19)	31941 (28990, 35419)	32071 (28855, 36153)	−0.19 (−0.22, −0.16)	316 (177, 552)	308 (167, 548)	−0.22 (−0.25, −0.19)
			Andorra	0 (0, 0)	0 (0, 0)	−0.05 (−0.09, −0.02)	4 (4, 5)	7 (7, 8)	−0.07 (−0.08, −0.05)	0 (0, 0)	0 (0, 0)	−0.06 (−0.09, −0.03)
			Austria	17 (13, 23)	12 (9, 16)	−0.33 (−0.38, −0.29)	817 (742, 906)	738 (666, 831)	−0.29 (−0.32, −0.25)	8 (4, 14)	7 (4, 13)	−0.28 (−0.32, −0.25)
			Belgium	17 (13, 22)	16 (12, 21)	−0.14 (−0.18, −0.1)	875 (789, 986)	940 (844, 1058)	−0.13 (−0.15, −0.12)	9 (5, 15)	9 (5, 16)	−0.15 (−0.18, −0.12)
			Cyprus	2 (1, 2)	2 (1, 2)	−0.23 (−0.28, −0.17)	62 (56, 69)	104 (94, 118)	−0.18 (−0.2, −0.14)	1 (0, 1)	1 (1, 2)	−0.24 (−0.31, −0.2)
			Denmark	8 (6, 10)	7 (5, 9)	−0.18 (−0.22, −0.13)	401 (363, 447)	415 (371, 471)	−0.13 (−0.16, −0.09)	4 (2, 7)	4 (2, 7)	−0.17 (−0.23, −0.11)
			Finland	10 (8, 13)	8 (6, 11)	−0.19 (−0.23, −0.15)	505 (449, 569)	527 (469, 603)	−0.14 (−0.17, −0.11)	5 (3, 9)	5 (3, 9)	−0.19 (−0.24, −0.15)
			France	109 (85, 140)	93 (71, 120)	−0.2 (−0.24, −0.16)	5074 (4604, 5624)	5308 (4779, 5960)	−0.18 (−0.21, −0.15)	51 (29, 89)	51 (28, 92)	−0.21 (−0.25, −0.18)
			Germany	115 (89, 148)	97 (75, 126)	−0.13 (−0.17, −0.09)	6145 (5546, 6868)	6354 (5732, 7172)	−0.1 (−0.13, −0.08)	63 (35, 109)	61 (33, 108)	−0.16 (−0.21, −0.12)
			Greece	16 (13, 21)	12 (9, 16)	−0.15 (−0.2, −0.11)	852 (771, 949)	801 (725, 890)	−0.17 (−0.19, −0.14)	8 (5, 15)	8 (4, 13)	−0.19 (−0.23, −0.16)
			Iceland	0 (0, 1)	0 (0, 1)	−0.22 (−0.26, −0.17)	20 (18, 22)	26 (23, 29)	−0.18 (−0.21, −0.16)	0 (0, 0)	0 (0, 0)	−0.18 (−0.21, −0.14)
			Ireland	7 (5, 8)	6 (5, 8)	−0.21 (−0.26, −0.14)	274 (247, 306)	357 (319, 405)	−0.17 (−0.21, −0.14)	3 (1, 5)	3 (2, 6)	−0.18 (−0.22, −0.14)
			Israel	9 (7, 12)	14 (11, 17)	−0.19 (−0.24, −0.12)	346 (311, 390)	607 (540, 701)	−0.16 (−0.2, −0.09)	3 (2, 6)	6 (3, 11)	−0.16 (−0.22, −0.09)
			Italy	104 (80, 133)	65 (50, 83)	−0.31 (−0.36, −0.24)	5427 (4880, 6056)	4556 (4038, 5268)	−0.28 (−0.32, −0.23)	53 (29, 93)	45 (24, 79)	−0.27 (−0.31, −0.22)
			Luxembourg	1 (1, 1)	1 (1, 1)	−0.25 (−0.3, −0.21)	37 (34, 41)	52 (46, 58)	−0.22 (−0.24, −0.19)	0 (0, 1)	0 (0, 1)	−0.25 (−0.3, −0.22)
			Malta	1 (0, 1)	1 (0, 1)	−0.1 (−0.15, −0.06)	29 (26, 32)	38 (34, 43)	−0.07 (−0.09, −0.06)	0 (0, 1)	0 (0, 1)	−0.12 (−0.17, −0.08)
			Monaco	0 (0, 0)	0 (0, 0)	−0.05 (−0.09, −0.01)	2 (2, 3)	3 (3, 3)	−0.04 (−0.05, −0.02)	0 (0, 0)	0 (0, 0)	−0.04 (−0.07, −0.01)
			Netherlands	19 (14, 24)	18 (14, 24)	−0.09 (−0.13, −0.06)	919 (839, 1021)	1079 (983, 1206)	−0.09 (−0.11, −0.07)	9 (5, 16)	10 (5, 18)	−0.09 (−0.12, −0.06)
			Norway	8 (6, 11)	7 (5, 9)	−0.32 (−0.35, −0.28)	394 (359, 437)	382 (346, 426)	−0.28 (−0.3, −0.26)	4 (2, 7)	4 (2, 6)	−0.29 (−0.31, −0.26)
			Portugal	19 (15, 24)	9 (7, 12)	−0.44 (−0.48, −0.4)	907 (828, 1000)	647 (590, 724)	−0.43 (−0.44, −0.4)	10 (6, 16)	6 (3, 11)	−0.49 (−0.55, −0.45)
			San Marino	0 (0, 0)	0 (0, 0)	−0.08 (−0.12, −0.05)	2 (2, 2)	3 (3, 3)	−0.08 (−0.1, −0.06)	0 (0, 0)	0 (0, 0)	−0.08 (−0.11, −0.04)
			Spain	67 (52, 85)	50 (38, 64)	−0.25 (−0.31, −0.18)	3076 (2814, 3391)	3334 (2996, 3769)	−0.2 (−0.24, −0.14)	30 (16, 52)	32 (17, 57)	−0.2 (−0.24, −0.14)
			Sweden	14 (11, 18)	13 (9, 16)	−0.24 (−0.27, −0.21)	755 (685, 836)	737 (666, 828)	−0.22 (−0.24, −0.2)	7 (4, 13)	7 (4, 12)	−0.22 (−0.24, −0.19)
			Switzerland	18 (14, 23)	14 (10, 18)	−0.36 (−0.41, −0.31)	872 (784, 969)	831 (741, 939)	−0.32 (−0.35, −0.29)	8 (4, 15)	8 (4, 14)	−0.3 (−0.33, −0.27)
			United Kingdom	79 (61, 100)	70 (53, 89)	−0.22 (−0.24, −0.19)	4119 (3732, 4567)	4197 (3790, 4681)	−0.2 (−0.21, −0.18)	40 (22, 71)	40 (21, 71)	−0.22 (−0.25, −0.2)
	Latin America and Caribbean			1132(932,1385)	1188(973,1436)	−0.26 (−0.28, −0.23)	33055 (30077, 36592)	49068 (45013, 53828)	−0.23 (−0.24, −0.22)	499 (353, 723)	632 (426, 957)	−0.35 (−0.41, −0.3)
		Southern Latin America		149(110,199)	174 (129, 234)	−0.1 (−0.14, −0.05)	5355 (4822, 5988)	7726 (6998, 8610)	−0.09 (−0.1, −0.07)	54 (32, 91)	70 (39, 126)	−0.17 (−0.24, −0.12)
			Argentina	100 (74, 135)	114 (84, 152)	−0.14 (−0.18, −0.09)	3660 (3289, 4121)	4927 (4442, 5488)	−0.13 (−0.15, −0.11)	37 (22, 63)	46 (26, 81)	−0.19 (−0.24, −0.15)
			Chile	35 (26, 47)	50 (37, 67)	0.12 (0.04, 0.2)	1172 (1062, 1304)	2328 (2101, 2600)	0.13 (0.09, 0.16)	12 (7, 20)	20 (10, 38)	−0.05 (−0.2, 0.05)
			Uruguay	14 (10, 19)	10 (7, 14)	−0.28 (−0.35, −0.2)	523 (468, 588)	471 (427, 524)	−0.26 (−0.28, −0.24)	5 (3, 9)	4 (2, 8)	−0.28 (−0.33, −0.25)
		High-income North America		451(346,572)	390 (308, 490)	−0.34 (−0.37, −0.31)	19677 (18106, 21680)	18780 (17391, 20367)	−0.37 (−0.39, −0.35)	187 (102, 331)	183 (103, 315)	−0.36 (−0.38, −0.34)
			Canada	38 (30, 49)	38 (29, 48)	−0.25 (−0.29, −0.21)	1594 (1467, 1755)	1936 (1785, 2121)	−0.25 (−0.27, −0.23)	15 (8, 27)	19 (10, 32)	−0.24 (−0.27, −0.21)
			Greenland	0 (0, 0)	0 (0, 0)	−0.29 (−0.33, −0.25)	4 (3, 4)	3 (3, 4)	−0.28 (−0.3, −0.26)	0 (0, 0)	0 (0, 0)	−0.38 (−0.44, −0.33)
			United States of America	412 (316, 523)	352 (279, 442)	−0.35 (−0.38, −0.32)	18078 (16620, 19924)	16841 (15611, 18246)	−0.38 (−0.4, −0.36)	172 (94, 303)	165 (92, 282)	−0.37 (−0.39, −0.35)
		Caribbean		71 (58, 87)	107 (88, 130)	0.18 (0.13, 0.26)	2304 (2134, 2512)	4061 (3751, 4420)	0.1 (0.07, 0.14)	31 (21, 45)	56 (39, 84)	0.13 (0.02, 0.33)
			Antigua and Barbuda	0 (0, 0)	0 (0, 0)	0.07 (−0.02, 0.16)	6 (5, 6)	11 (10, 12)	0.09 (0.06, 0.12)	0 (0, 0)	0 (0, 0)	−0.05 (−0.13, 0.03)
			Bahamas	0 (0, 1)	1 (0, 1)	0 (−0.04, 0.04)	13 (12, 15)	26 (24, 28)	0.05 (0.02, 0.08)	0 (0, 0)	0 (0, 0)	−0.06 (−0.13, 0.01)
			Barbados	0 (0, 0)	0 (0, 1)	0.01 (−0.04, 0.06)	16 (14, 17)	22 (21, 25)	0.01 (−0.01, 0.03)	0 (0, 0)	0 (0, 0)	−0.12 (−0.22, −0.06)
			Belize	0 (0, 1)	1 (1, 1)	0.05 (−0.05, 0.14)	11 (10, 12)	34 (31, 38)	0.12 (0.09, 0.16)	0 (0, 0)	0 (0, 1)	0.04 (−0.02, 0.09)
			Bermuda	0 (0, 0)	0 (0, 0)	−0.04 (−0.08, 0.01)	4 (4, 5)	5 (5, 6)	−0.01 (−0.03, 0.02)	0 (0, 0)	0 (0, 0)	−0.23 (−0.37, −0.12)
			Cuba	22 (18, 26)	22 (18, 26)	0.06 (0.01, 0.1)	812 (752, 883)	1098 (1013, 1194)	0.05 (0.02, 0.07)	10 (6, 15)	11 (7, 19)	−0.14 (−0.26, −0.06)
			Dominica	0 (0, 0)	0 (0, 0)	0.04 (−0.01, 0.1)	4 (3, 4)	5 (4, 5)	0.11 (0.08, 0.14)	0 (0, 0)	0 (0, 0)	0.02 (−0.05, 0.09)
			Dominican Republic	12 (10, 15)	21 (17, 26)	0.22 (0.16, 0.29)	342 (316, 373)	772 (715, 840)	0.17 (0.14, 0.19)	5 (3, 7)	10 (7, 15)	0.01 (−0.08, 0.08)
			Grenada	0 (0, 0)	0 (0, 0)	0.07 (−0.02, 0.16)	7 (6, 7)	10 (9, 11)	0 (−0.05, 0.05)	0 (0, 0)	0 (0, 0)	−0.23 (−0.33, −0.12)
			Guyana	1 (1, 2)	2 (1, 2)	0.16 (0.1, 0.21)	41 (38, 45)	55 (51, 60)	0.11 (0.09, 0.14)	1 (0, 1)	1 (1, 1)	0 (−0.06, 0.06)
			Haiti	16 (13, 20)	40 (32, 50)	0.22 (0.1, 0.41)	427 (394, 465)	1151 (1027, 1309)	0.15 (0.05, 0.28)	7 (5, 9)	23 (16, 35)	0.4 (0.13, 0.89)
			Jamaica	6 (5, 8)	7 (5, 9)	−0.04 (−0.12, 0.03)	178 (163, 195)	244 (224, 268)	−0.1 (−0.11, −0.08)	2 (1, 3)	3 (2, 4)	−0.15 (−0.21, −0.11)
			Puerto Rico	6 (5, 8)	6 (5, 7)	0.08 (0.03, 0.13)	254 (232, 277)	308 (282, 338)	0.03 (0, 0.07)	3 (2, 5)	3 (2, 5)	−0.19 (−0.34, −0.08)
			Saint Kitts and Nevis	0 (0, 0)	0 (0, 0)	0.03 (−0.06, 0.11)	3 (3, 4)	7 (6, 7)	0.09 (0.05, 0.13)	0 (0, 0)	0 (0, 0)	−0.12 (−0.23, −0.02)
			Saint Lucia	0 (0, 0)	0 (0, 0)	0.05 (−0.02, 0.13)	7 (7, 8)	15 (14, 17)	0.09 (0.06, 0.11)	0 (0, 0)	0 (0, 0)	−0.1 (−0.19, −0.02)
			Saint Vincent and theGrenadines	0 (0, 0)	0 (0, 0)	0.07 (0, 0.14)	6 (6, 7)	10 (9, 11)	0.08 (0.05, 0.11)	0 (0, 0)	0 (0, 0)	−0.03 (−0.1, 0.02)
			Suriname	1 (1, 1)	1 (1, 1)	0.06 (0.01, 0.12)	23 (20, 33)	41 (37, 50)	−0.01 (−0.11, 0.03)	0 (0, 1)	1 (0, 1)	−0.16 (−0.29, −0.07)
			Trinidad and Tobago	2 (2, 3)	2 (2, 3)	0 (−0.06, 0.07)	66 (60, 72)	102 (94, 112)	0.03 (0, 0.07)	1 (1, 1)	1 (1, 2)	−0.06 (−0.14, 0.02)
			United States Virgin Islands	0 (0, 0)	0 (0, 0)	−0.07 (−0.11, −0.03)	7 (7, 8)	7 (7, 8)	−0.1 (−0.12, −0.08)	0 (0, 0)	0 (0, 0)	−0.23 (−0.3, −0.17)
		Andean Latin America		105(86,130)	118 (96, 146)	−0.31 (−0.42, −0.23)	2464 (2246, 2732)	4526 (4155, 4955)	−0.13 (−0.15, −0.11)	39 (27, 55)	54 (36, 85)	−0.34 (−0.42, −0.25)
			Bolivia (Plurinational State of)	13 (11, 16)	21 (16, 26)	−0.15 (−0.2, −0.1)	358 (332, 389)	685 (636, 744)	−0.17 (−0.18, −0.15)	6 (4, 8)	9 (6, 13)	−0.3 (−0.36, −0.24)
			Ecuador	20 (17, 25)	32 (26, 39)	−0.1 (−0.15, −0.06)	596 (554, 647)	1188 (1101, 1300)	−0.11 (−0.13, −0.09)	9 (6, 13)	15 (10, 23)	−0.27 (−0.35, −0.19)
			Peru	71 (57, 91)	66 (53, 82)	−0.41 (−0.53, −0.29)	1509 (1347, 1736)	2654 (2416, 2952)	−0.13 (−0.16, −0.1)	24 (16, 35)	30 (19, 49)	−0.37 (−0.48, −0.27)
		Central Latin America		583(472,726)	543 (436, 677)	−0.34 (−0.37, −0.32)	16705 (15044, 18708)	22815 (20713, 25324)	−0.31 (−0.31, −0.29)	253 (176, 364)	285 (189, 438)	−0.43 (−0.49, −0.38)
			Colombia	121 (99, 150)	92 (75, 113)	−0.43 (−0.47, −0.39)	3380 (3063, 3750)	4457 (4000, 4991)	−0.32 (−0.35, −0.28)	48 (33, 70)	52 (33, 83)	−0.45 (−0.53, −0.38)
			Costa Rica	7 (5, 9)	8 (7, 10)	−0.11 (−0.14, −0.08)	212 (188, 241)	379 (341, 426)	−0.11 (−0.12, −0.09)	3 (2, 4)	4 (2, 7)	−0.24 (−0.33, −0.17)
			El Salvador	25 (19, 34)	12 (10, 15)	−0.53 (−0.64, −0.39)	755 (582, 1016)	677 (567, 832)	−0.3 (−0.35, −0.26)	20 (12, 31)	11 (7, 17)	−0.54 (−0.61, −0.46)
			Guatemala	33 (25, 43)	50 (40, 64)	−0.12 (−0.33, 0.06)	853 (711, 1079)	1763 (1570, 2008)	−0.04 (−0.11, 0.02)	18 (11, 29)	25 (17, 37)	−0.34 (−0.43, −0.22)
			Honduras	13 (11, 17)	22 (18, 27)	−0.17 (−0.2, −0.13)	339 (305, 377)	732 (667, 815)	−0.17 (−0.2, −0.14)	5 (4, 7)	10 (7, 15)	−0.2 (−0.25, −0.15)
			Mexico	307 (241, 384)	271 (216, 335)	−0.37 (−0.39, −0.35)	8680 (7853, 9691)	10741 (9800, 11841)	−0.38 (−0.39, −0.37)	120 (83, 176)	131 (86, 202)	−0.47 (−0.52, −0.43)
			Nicaragua	10 (8, 13)	12 (9, 15)	−0.23 (−0.28, −0.19)	502 (371, 708)	627 (518, 787)	−0.32 (−0.37, −0.27)	13 (7, 22)	10 (6, 17)	−0.51 (−0.57, −0.43)
			Panama	5 (4, 7)	8 (6, 10)	−0.13 (−0.16, −0.1)	186 (164, 213)	336 (301, 378)	−0.13 (−0.16, −0.1)	2 (2, 4)	4 (2, 6)	−0.29 (−0.39, −0.21)
			Venezuela (Bolivarian Republic of)	62 (49, 78)	68 (54, 86)	−0.09 (−0.14, −0.04)	1797 (1608, 2011)	3101 (2824, 3414)	−0.05 (−0.08, −0.02)	24 (16, 35)	37 (24, 58)	−0.17 (−0.24, −0.11)
		Tropical Latin America		373(303,463)	420 (345, 510)	−0.19 (−0.22, −0.16)	11582 (10396, 13003)	17666 (16008, 19673)	−0.21 (−0.23, −0.18)	177 (125, 253)	237 (160, 355)	−0.33 (−0.39, −0.27)
			Brazil	365 (296, 453)	407 (334, 493)	−0.2 (−0.23, −0.16)	11342 (10178, 12735)	17208 (15586, 19181)	−0.21 (−0.23, −0.18)	174 (123, 248)	232 (156, 346)	−0.33 (−0.39, −0.27)
			Paraguay	8 (7, 10)	13 (11, 16)	−0.07 (−0.11, −0.03)	240 (215, 273)	458 (417, 510)	−0.13 (−0.15, −0.11)	3 (2, 5)	6 (4, 9)	−0.21 (−0.27, −0.16)
	North Africa and Middle East			869(742,1016)	1463(1208,1767)	−0.05 (−0.15, 0.08)	24093 (21583, 27696)	48250 (42179, 56635)	−0.11 (−0.15, −0.05)	445 (301, 690)	720 (472, 1107)	−0.26 (−0.36, −0.18)
		North Africa and Middle East		869(742,1016)	1463 (1208, 1767)	−0.05 (−0.15, 0.08)	24093 (21583, 27696)	48250 (42179, 56635)	−0.11 (−0.15, −0.05)	445 (301, 690)	720 (472, 1107)	−0.26 (−0.36, −0.18)
			Afghanistan	30 (24, 39)	298 (196, 444)	2.2 (1.37, 3.16)	1802 (870, 3591)	4413 (2888, 6681)	−0.04 (−0.28, 0.41)	67 (20, 191)	122 (64, 229)	−0.25 (−0.46, 0.4)
			Algeria	53 (44, 64)	61 (51, 74)	−0.3 (−0.34, −0.26)	1427 (1331, 1544)	2489 (2286, 2728)	−0.25 (−0.28, −0.21)	22 (15, 32)	31 (20, 48)	−0.4 (−0.48, −0.33)
			Bahrain	1 (1, 1)	2 (2, 2)	−0.09 (−0.13, −0.04)	25 (23, 27)	89 (82, 98)	−0.08 (−0.1, −0.05)	0 (0, 1)	1 (1, 2)	−0.36 (−0.49, −0.24)
			Egypt	99 (82, 119)	138 (115, 168)	−0.24 (−0.28, −0.19)	2822 (2600, 3058)	4459 (4075, 4960)	−0.24 (−0.27, −0.18)	46 (33, 64)	61 (41, 94)	−0.36 (−0.43, −0.28)
			Iran (Islamic Republic of)	184 (154, 226)	139 (115, 167)	−0.47 (−0.55, −0.39)	4494 (3997, 5155)	6399 (5819, 7108)	−0.31 (−0.34, −0.29)	80 (55, 115)	78 (49, 124)	−0.53 (−0.61, −0.44)
			Iraq	82 (67, 100)	127 (103, 155)	−0.32 (−0.37, −0.26)	2817 (2367, 3358)	6570 (5251, 8406)	−0.09 (−0.16, −0.01)	68 (43, 104)	107 (65, 170)	−0.32 (−0.4, −0.23)
			Jordan	6 (5, 7)	14 (12, 18)	−0.26 (−0.3, −0.22)	155 (142, 170)	515 (470, 566)	−0.24 (−0.26, −0.22)	2 (1, 3)	5 (3, 9)	−0.41 (−0.5, −0.33)
			Kuwait	11 (8, 16)	6 (5, 8)	−0.79 (−0.86, −0.67)	101 (92, 111)	332 (295, 380)	−0.09 (−0.13, −0.04)	1 (1, 2)	3 (2, 6)	−0.23 (−0.34, −0.15)
			Lebanon	12 (9, 18)	7 (6, 9)	−0.66 (−0.75, −0.55)	401 (273, 636)	510 (384, 734)	−0.4 (−0.46, −0.34)	10 (5, 21)	7 (4, 13)	−0.66 (−0.74, −0.54)
			Libya	8 (6, 9)	12 (10, 14)	0.05 (−0.07, 0.19)	231 (212, 254)	630 (538, 752)	0.2 (0.08, 0.36)	3 (2, 5)	9 (6, 14)	0.16 (0.02, 0.3)
			Morocco	56 (46, 68)	61 (49, 73)	−0.24 (−0.28, −0.2)	1653 (1537, 1789)	2298 (2126, 2490)	−0.26 (−0.29, −0.24)	25 (18, 36)	30 (20, 45)	−0.37 (−0.42, −0.31)
			Palestine	7 (5, 10)	10 (8, 12)	−0.44 (−0.52, −0.35)	190 (146, 256)	566 (423, 783)	0.15 (−0.01, 0.3)	5 (3, 7)	10 (6, 15)	−0.14 (−0.27, −0.01)
			Oman	5 (4, 6)	8 (7, 10)	−0.32 (−0.36, −0.26)	133 (124, 143)	295 (270, 325)	−0.29 (−0.32, −0.27)	2 (1, 3)	3 (2, 5)	−0.5 (−0.59, −0.41)
			Qatar	1 (1, 2)	7 (5, 8)	−0.24 (−0.29, −0.18)	36 (33, 40)	238 (214, 265)	−0.22 (−0.25, −0.19)	0 (0, 1)	2 (1, 4)	−0.43 (−0.55, −0.33)
			Saudi Arabia	59 (48, 73)	140 (112, 176)	−0.08 (−0.15, 0)	1523 (1393, 1667)	4730 (4249, 5286)	−0.09 (−0.13, −0.05)	21 (14, 31)	51 (31, 83)	−0.29 (−0.4, −0.2)
			Sudan	75 (57, 103)	68 (57, 82)	−0.57 (−0.68, −0.45)	1085 (978, 1225)	2459 (2097, 2958)	0 (−0.08, 0.1)	19 (14, 27)	40 (26, 59)	−0.07 (−0.17, 0.02)
			Syrian Arab Republic	19 (16, 24)	28 (22, 36)	0.4 (0.14, 0.8)	570 (490, 719)	2085 (1506, 2947)	1.48 (0.89, 2.27)	9 (6, 15)	39 (22, 65)	1.95 (1.12, 3.09)
			Tunisia	21 (17, 27)	23 (18, 29)	−0.21 (−0.26, −0.16)	628 (577, 689)	933 (862, 1024)	−0.23 (−0.25, −0.21)	8 (5, 12)	10 (6, 16)	−0.37 (−0.46, −0.3)
			Turkey	109 (90, 131)	107 (88, 131)	−0.27 (−0.33, −0.22)	3193 (2946, 3476)	4853 (4473, 5325)	−0.2 (−0.23, −0.16)	44 (30, 65)	52 (31, 86)	−0.39 (−0.49, −0.3)
			United Arab Emirates	5 (4, 6)	20 (16, 25)	−0.17 (−0.21, −0.12)	140 (129, 152)	925 (835, 1026)	−0.13 (−0.15, −0.1)	2 (1, 3)	11 (7, 17)	−0.29 (−0.38, −0.22)
			Yemen	24 (20, 29)	184 (124, 272)	1.96 (0.98, 3.64)	651 (592, 737)	2417 (1928, 3075)	0.2 (0.06, 0.4)	12 (9, 17)	48 (32, 74)	0.25 (0.07, 0.43)
	South Asia			1547(1291,1885)	1906 (1588, 2284)	−0.25 (−0.3, −0.21)	41490 (37629, 46287)	68908 (62351, 77093)	−0.19 (−0.21, −0.16)	769 (568, 1037)	1180 (848, 1638)	−0.27 (−0.32, −0.23)
		South Asia		1547 (1291, 1885)	1906 (1588, 2284)	−0.25 (−0.3, −0.21)	41490 (37629, 46287)	68908 (62351, 77093)	−0.19 (−0.21, −0.16)	769 (568, 1037)	1180 (848, 1638)	−0.27 (−0.32, −0.23)
			Bangladesh	94 (79, 111)	118 (96, 143)	−0.1 (−0.16, −0.04)	2482 (2269, 2736)	5148 (4594, 5794)	0.04 (−0.01, 0.11)	45 (33, 62)	76 (53, 111)	−0.18 (−0.28, −0.07)
			Bhutan	1 (1, 1)	1 (1, 1)	0.07 (0, 0.16)	21 (20, 23)	37 (34, 40)	0.04 (0.01, 0.07)	0 (0, 0)	0 (0, 1)	−0.11 (−0.19, −0.03)
			India	1326 (1100, 1631)	1553 (1282, 1880)	−0.28 (−0.34, −0.23)	35658 (32211, 39959)	55389 (50126, 61979)	−0.23 (−0.25, −0.21)	663 (488, 898)	946 (676, 1313)	−0.32 (−0.36, −0.27)
			Nepal	24 (19, 29)	35 (28, 44)	−0.05 (−0.1, 0)	670 (599, 750)	1470 (1281, 1699)	0.11 (0.03, 0.22)	12 (9, 17)	26 (18, 36)	0.04 (−0.08, 0.18)
			Pakistan	101 (85, 120)	200 (170, 233)	−0.07 (−0.1, −0.02)	2660 (2461, 2903)	6864 (6080, 7876)	0.05 (−0.02, 0.16)	48 (35, 66)	132 (92, 183)	0.08 (−0.03, 0.21)
	Sub-Saharan Africa			1027(816,1321)	1267 (1059, 1533)	−0.44 (−0.53, −0.36)	17494 (15645, 19967)	35257 (31163, 41030)	−0.12 (−0.15, −0.07)	353 (246, 510)	672 (460, 986)	−0.13 (−0.18, −0.07)
		Central Sub-Saharan Africa		78(65,95)	150 (127, 178)	−0.2 (−0.24, −0.16)	1855 (1648, 2117)	4568 (3860, 5590)	0.02 (−0.07, 0.14)	39 (27, 54)	95 (64, 139)	0.03 (−0.07, 0.15)
			Angola	31 (22, 43)	29 (24, 35)	−0.68 (−0.77, −0.58)	669 (511, 898)	1305 (1032, 1713)	−0.19 (−0.23, −0.16)	18 (11, 27)	28 (18, 43)	−0.29 (−0.34, −0.24)
			Central African Republic	3 (2, 4)	14 (11, 19)	1.25 (0.7, 2.17)	76 (70, 81)	226 (190, 280)	0.29 (0.13, 0.54)	1 (1, 2)	6 (4, 8)	0.56 (0.31, 0.9)
			Congo	2 (2, 3)	4 (4, 5)	−0.19 (−0.22, −0.15)	63 (58, 68)	205 (170, 258)	0.23 (0.04, 0.52)	1 (1, 2)	5 (3, 7)	0.43 (0.1, 0.89)
			Democratic Republic of the Congo	41 (34, 49)	100 (84, 119)	0.07 (−0.02, 0.22)	1004 (920, 1089)	2751 (2367, 3278)	0.09 (−0.03, 0.28)	18 (13, 24)	55 (38, 79)	0.2 (0.03, 0.45)
			Equatorial Guinea	0 (0, 1)	1 (1, 1)	−0.28 (−0.31, −0.23)	12 (11, 13)	32 (29, 35)	−0.3 (−0.31, −0.28)	0 (0, 0)	0 (0, 1)	−0.44 (−0.5, −0.37)
			Gabon	1 (1, 1)	2 (1, 2)	−0.18 (−0.21, −0.15)	30 (28, 32)	48 (45, 52)	−0.19 (−0.2, −0.18)	1 (0, 1)	1 (1, 1)	−0.28 (−0.33, −0.23)
		Eastern Sub-Saharan Africa		631(460,883)	520 (420, 653)	−0.61 (−0.7, −0.51)	7734 (6565, 9567)	14681 (12496, 18077)	−0.11 (−0.14, −0.06)	176 (117, 267)	308 (204, 471)	−0.11 (−0.17, −0.05)
			Burundi	6 (5, 8)	14 (12, 17)	−0.06 (−0.11, 0.01)	156 (143, 168)	1124 (810, 1603)	1.91 (1.12, 2.98)	3 (2, 4)	35 (21, 55)	4.05 (2.36, 6.64)
			Comoros	1 (0, 1)	1 (1, 1)	−0.14 (−0.17, −0.11)	13 (12, 14)	24 (22, 26)	−0.11 (−0.13, −0.08)	0 (0, 0)	0 (0, 1)	−0.14 (−0.2, −0.09)
			Djibouti	1 (1, 1)	1 (1, 1)	−0.45 (−0.58, −0.32)	12 (11, 13)	44 (40, 48)	−0.02 (−0.06, 0.05)	0 (0, 0)	1 (0, 1)	−0.04 (−0.12, 0.07)
			Eritrea	53 (33, 83)	7 (6, 8)	−0.93 (−0.96, −0.88)	550 (367, 810)	560 (395, 838)	−0.3 (−0.35, −0.22)	22 (13, 38)	17 (10, 30)	−0.43 (−0.49, −0.32)
			Ethiopia	372 (247, 553)	193 (137, 297)	−0.76 (−0.83, −0.66)	2191 (1950, 2528)	3681 (3129, 4799)	−0.22 (−0.28, −0.07)	47 (33, 66)	67 (44, 108)	−0.3 (−0.4, −0.1)
			Kenya	25 (20, 31)	47 (39, 56)	−0.09 (−0.11, −0.06)	603 (548, 652)	1371 (1260, 1483)	−0.11 (−0.13, −0.08)	9 (7, 13)	22 (16, 31)	−0.1 (−0.13, −0.07)
			Madagascar	13 (11, 16)	26 (22, 31)	−0.16 (−0.2, −0.12)	345 (316, 372)	674 (619, 731)	−0.23 (−0.24, −0.21)	6 (4, 8)	10 (7, 15)	−0.24 (−0.28, −0.2)
			Malawi	10 (8, 12)	16 (13, 19)	−0.15 (−0.18, −0.11)	253 (229, 274)	422 (385, 460)	−0.19 (−0.2, −0.17)	4 (3, 6)	7 (5, 9)	−0.22 (−0.27, −0.17)
			Mozambique	27 (19, 40)	36 (30, 43)	−0.41 (−0.55, −0.24)	910 (634, 1433)	1104 (900, 1440)	−0.28 (−0.34, −0.21)	27 (15, 49)	25 (16, 39)	−0.4 (−0.46, −0.31)
			Rwanda	26 (18, 39)	11 (9, 13)	−0.75 (−0.83, −0.63)	209 (192, 225)	825 (638, 1063)	0.99 (0.55, 1.56)	4 (3, 5)	32 (19, 54)	2.97 (1.44, 5.04)
			Somalia	27 (19, 41)	40 (32, 50)	−0.46 (−0.57, −0.32)	296 (227, 545)	985 (790, 1338)	0.25 (0.01, 0.51)	7 (4, 18)	23 (15, 36)	0.35 (−0.12, 0.8)
			South Sudan	7 (6, 8)	16 (13, 20)	0.4 (0.2, 0.71)	252 (215, 302)	493 (392, 647)	0.26 (0.13, 0.42)	5 (3, 7)	10 (7, 16)	0.35 (0.2, 0.5)
			United Republic of Tanzania	29 (24, 35)	54 (45, 65)	−0.13 (−0.16, −0.1)	715 (649, 773)	1491 (1358, 1621)	−0.14 (−0.15, −0.12)	12 (8, 16)	23 (16, 33)	−0.17 (−0.22, −0.13)
			Uganda	24 (20, 29)	40 (33, 48)	−0.3 (−0.4, −0.22)	1014 (723, 1495)	1405 (1156, 1775)	−0.27 (−0.32, −0.22)	26 (15, 45)	27 (18, 43)	−0.38 (−0.44, −0.31)
			Zambia	9 (7, 10)	18 (15, 21)	−0.1 (−0.15, −0.06)	207 (188, 223)	464 (423, 506)	−0.13 (−0.14, −0.11)	4 (3, 5)	7 (5, 10)	−0.17 (−0.22, −0.13)
		Southern Sub-Saharan Africa		79(66,93)	87 (73, 103)	−0.28 (−0.3, −0.25)	2295 (2106, 2548)	2981 (2761, 3234)	−0.29 (−0.3, −0.28)	43 (31, 60)	51 (36, 71)	−0.37 (−0.4, −0.34)
			Botswana	2 (1, 2)	3 (2, 3)	−0.02 (−0.07, 0.04)	38 (35, 41)	90 (83, 96)	−0.01 (−0.03, 0.02)	1 (0, 1)	1 (1, 2)	−0.04 (−0.09, 0.02)
			Lesotho	2 (1, 2)	2 (2, 3)	0.21 (0.15, 0.28)	43 (39, 46)	68 (64, 73)	0.18 (0.16, 0.21)	1 (1, 1)	1 (1, 2)	0.26 (0.19, 0.33)
			Namibia	2 (1, 2)	3 (2, 3)	−0.06 (−0.1, −0.02)	71 (57, 92)	96 (86, 110)	−0.21 (−0.26, −0.17)	2 (1, 2)	2 (1, 2)	−0.37 (−0.44, −0.3)
			South Africa	63 (52, 75)	64 (53, 75)	−0.34 (−0.36, −0.31)	1870 (1712, 2101)	2302 (2134, 2517)	−0.35 (−0.36, −0.33)	35 (25, 49)	39 (27, 55)	−0.43 (−0.46, −0.4)
			Eswatini	1 (1, 1)	2 (1, 2)	0.3 (0.12, 0.61)	23 (21, 25)	38 (35, 41)	−0.06 (−0.08, −0.04)	0 (0, 1)	1 (0, 1)	−0.12 (−0.17, −0.08)
			Zimbabwe	10 (8, 12)	14 (12, 17)	−0.06 (−0.09, −0.02)	251 (231, 271)	387 (359, 416)	−0.08 (−0.09, −0.07)	4 (3, 6)	7 (5, 9)	−0.05 (−0.09, 0)
		Western Sub-Saharan Africa		240(202,286)	510 (432, 612)	−0.14 (−0.17, −0.11)	5610 (5113, 6045)	13027 (11739, 14395)	−0.09 (−0.12, −0.04)	95 (70, 131)	219 (157, 304)	−0.09 (−0.14, −0.04)
			Benin	6 (5, 7)	14 (12, 17)	−0.06 (−0.1, −0.02)	134 (122, 144)	342 (313, 371)	−0.11 (−0.12, −0.09)	2 (2, 3)	5 (4, 7)	−0.16 (−0.21, −0.11)
			Burkina Faso	10 (8, 12)	31 (26, 39)	0.33 (0.15, 0.63)	247 (225, 269)	595 (540, 651)	−0.02 (−0.04, 0)	4 (3, 6)	10 (8, 14)	−0.04 (−0.09, 0.01)
			Cameroon	10 (8, 12)	36 (30, 43)	0.17 (0.06, 0.34)	264 (240, 288)	841 (757, 934)	−0.01 (−0.04, 0.03)	4 (3, 6)	14 (10, 20)	−0.04 (−0.1, 0.03)
			Cabo Verde	0 (0, 1)	1 (0, 1)	−0.07 (−0.11, −0.03)	11 (11, 12)	20 (19, 22)	−0.12 (−0.14, −0.1)	0 (0, 0)	0 (0, 0)	−0.23 (−0.29, −0.17)
			Chad	11 (9, 15)	26 (21, 31)	−0.21 (−0.28, −0.13)	254 (211, 318)	581 (504, 686)	−0.01 (−0.03, 0.01)	6 (4, 9)	11 (8, 16)	−0.09 (−0.14, −0.03)
			Côte d'Ivoire	13 (11, 16)	27 (22, 32)	−0.09 (−0.12, −0.06)	327 (297, 354)	775 (706, 849)	−0.07 (−0.09, −0.04)	5 (4, 7)	12 (9, 17)	−0.11 (−0.16, −0.06)
			Gambia	1 (1, 1)	2 (2, 3)	−0.09 (−0.12, −0.05)	29 (26, 33)	59 (54, 65)	−0.16 (−0.19, −0.14)	1 (0, 1)	1 (1, 1)	−0.22 (−0.28, −0.17)
			Ghana	14 (11, 17)	29 (24, 35)	−0.04 (−0.08, 0)	358 (326, 388)	847 (776, 926)	−0.07 (−0.09, −0.05)	6 (4, 8)	13 (9, 18)	−0.14 (−0.2, −0.08)
			Guinea	7 (6, 9)	14 (12, 17)	−0.04 (−0.08, 0)	187 (171, 202)	383 (347, 415)	−0.04 (−0.07, −0.01)	3 (2, 4)	6 (5, 9)	−0.03 (−0.09, 0.04)
			Guinea-Bissau	1 (1, 2)	2 (2, 2)	−0.18 (−0.21, −0.16)	32 (29, 34)	56 (51, 61)	−0.15 (−0.18, −0.1)	1 (0, 1)	1 (1, 1)	−0.13 (−0.19, −0.06)
			Liberia	23 (15, 34)	4 (4, 5)	−0.9 (−0.94, −0.85)	81 (73, 90)	254 (200, 332)	0.41 (0.17, 0.71)	2 (1, 2)	6 (4, 8)	0.7 (0.35, 1.1)
			Mali	14 (12, 17)	33 (28, 39)	−0.16 (−0.22, −0.1)	259 (236, 281)	858 (743, 1008)	0.26 (0.13, 0.46)	5 (3, 6)	16 (11, 23)	0.33 (0.14, 0.6)
			Mauritania	2 (2, 3)	4 (3, 4)	−0.24 (−0.27, −0.2)	58 (54, 63)	105 (96, 114)	−0.19 (−0.2, −0.17)	1 (1, 1)	2 (1, 2)	−0.32 (−0.38, −0.27)
			Niger	11 (9, 13)	32 (27, 40)	−0.01 (−0.06, 0.05)	223 (202, 245)	622 (561, 690)	−0.09 (−0.12, −0.05)	4 (3, 5)	11 (8, 15)	−0.11 (−0.16, −0.05)
			Nigeria	99 (82, 120)	226 (191, 272)	−0.1 (−0.15, −0.03)	2727 (2488, 2935)	5710 (5158, 6273)	−0.17 (−0.2, −0.14)	45 (33, 63)	93 (67, 131)	−0.18 (−0.22, −0.14)
			Sao Tome and Principe	0 (0, 0)	0 (0, 0)	0.13 (0.05, 0.27)	4 (4, 5)	7 (7, 8)	−0.07 (−0.1, −0.03)	0 (0, 0)	0 (0, 0)	−0.12 (−0.19, −0.07)
			Senegal	9 (7, 10)	14 (11, 17)	−0.16 (−0.21, −0.11)	197 (178, 213)	399 (363, 435)	−0.1 (−0.13, −0.07)	3 (2, 4)	6 (4, 9)	−0.13 (−0.2, −0.08)
			Sierra Leone	4 (4, 5)	8 (7, 9)	−0.11 (−0.14, −0.08)	121 (110, 132)	356 (297, 438)	0.41 (0.19, 0.73)	2 (1, 3)	7 (5, 11)	0.76 (0.37, 1.28)
			Togo	4 (3, 5)	7 (6, 9)	−0.11 (−0.14, −0.08)	95 (86, 103)	214 (196, 234)	−0.14 (−0.15, −0.12)	2 (1, 2)	4 (3, 5)	−0.15 (−0.2, −0.1)

### 3.2 Regional and national burden of disease from traumatic amputations

The burden of amputations and changes in the burden vary by region and country. In terms of number of all ages, South Asia has the highest incidence and YLDs, while the highest prevalence is in high-income countries. India and China were the top two countries with the highest incidence, prevalence and YLDs. The lowest regional, national burden of disease numbers are in Oceania and some of the countries in it located in the Western Pacific, such as Tokelau and Niue (Table 1).

**Figure 1:**
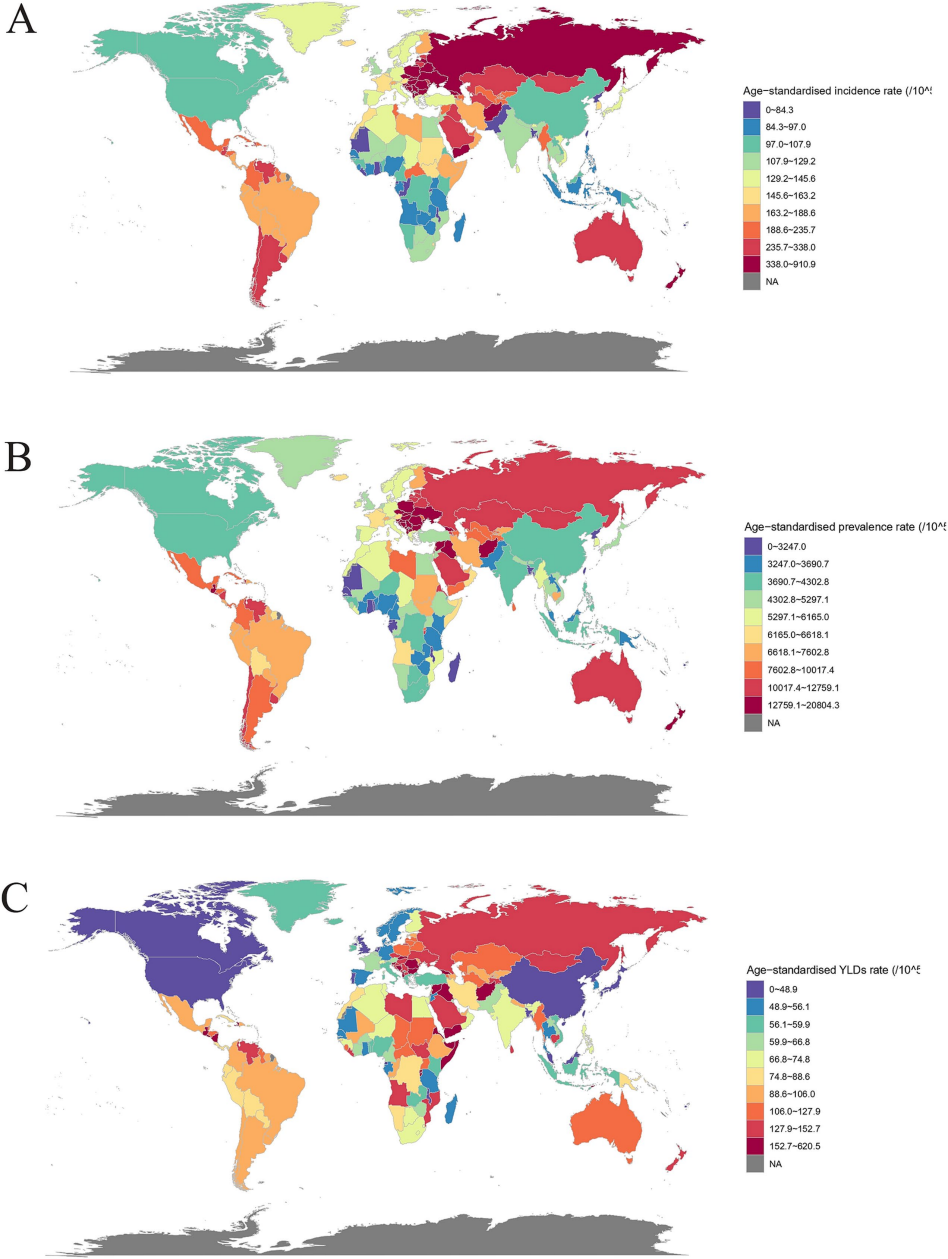
Global burden of traumatic amputations in countries and regions in 2021. **(A)** Age-standardized incidence rates; **(B)** Age-standardized prevalence rates; **(C)** Age-standardized YLDs rates.

When it comes to comparisons, it is important to look at the values of the rates (Figure 1). In 2021, the age-standardized incidence rate was highest in Central Europe (448.4 [95% UI 342.7–575.5] per 100,000) and lowest in East Asia (97.9 [79.1–121.5] per 100,000) (Supplementary Table 1). From 1990 to 2021, the Caribbean had the largest increase and Eastern Sub-Saharan Africa had the largest decrease in prevalence (Table 1). Age-standardized prevalence rates were highest in Central Europe (16129.1 [14704.0–17802.5] per 100,000) and lowest in Oceania (3542.4 [3224.1–3921.5] per 100,000) (Supplementary Table 2). From 1990 to 2021, Caribbean had the largest increase and High-income North America had the largest decrease (Table 1). Age-standardized YLDs rates were highest in Central Europe (145.3 [78.6–262.7] per 100,000) and lowest in High-income North America (38.8 [21.5–66.9] per 100,000) (Supplementary Table 1). From 1990 to 2021, Caribbean had the largest increase and East Asia had the largest decrease (Table 1). Note: The region to which a country belongs follows the rules for the classification of GBD results, as reflected in Table 1 and Supplementary Table 1.

The highest age-standardized incidence rates of traumatic amputation in 2021 were Afghanistan (910.9 [95% UI 596.8–1351.3] per 100,000), Albania (583.0 [439.3–759.7] per 100,000), Slovenia (572.2 [434.4–734.6] per 100,000). Kiribati (58.2 [49.8–68.2] per 100,000), Taiwan (Province of China) (59.3 [49.9–71.2] per 100,000), Tonga (61.2 [51.3–72.5] per 100,000) were the lowest (Figure 1A). From 1990 to 2021, the largest increases in age-standardized incidence rates were in Afghanistan, Yemen, and Central African Republic, and the largest decreases were in Eritrea, Liberia, and Kuwait (Table 1). The highest age-standardized prevalence of traumatic amputation was Afghanistan (20804.2 [13155.6–32892.7] per 100,000), Albania (20596.1 [18827.8–22760.6] per 100,000), Slovenia (20066. 9 [18261.0–22078.6] per 100,000). Taiwan (Province of China) (2203.8 [2035.0–2409.9] per 100,000), Democratic People’s Republic of Korea (2304.0 [2171.2–2465.4] per 100,000), Kiribati (2309.8 [2141.9–2516.6] per 100,000) were the lowest (Figure 1B). The largest rate of increase in age-standardized prevalence was in Burundi, Syrian Arab Republic, and Rwanda, and the largest rate of decrease was in Portugal, Taiwan (Province of China), and Lebanon (Table 1). The highest rates of age-standardized YLDs for traumatic amputations were Afghanistan (620.5 [303.9–1274.8] per 100,000), Eritrea (398.7 [226.9–681.2] per 100,000), Burundi (368.6 [224.6–579.7] per 100,000). Taiwan (Province of China) (25.8 [15.1–42.2] per 100,000), Tonga (37.0 [25.9–53.1] per 100,000), Canada (37.5 [25.9–53.1] per 100,000) were the lowest (Figure 1C). The largest increases in the rates of age-standardized YLDs were in Burundi, Rwanda, and Syrian Arab Republic, and the largest decreases were in Lebanon, El Salvador, and Taiwan (Province of China) (Table 1).

### 3.3 Regional, national, and SDI association analysis of the burden of traumatic amputation

**Figure 2:**
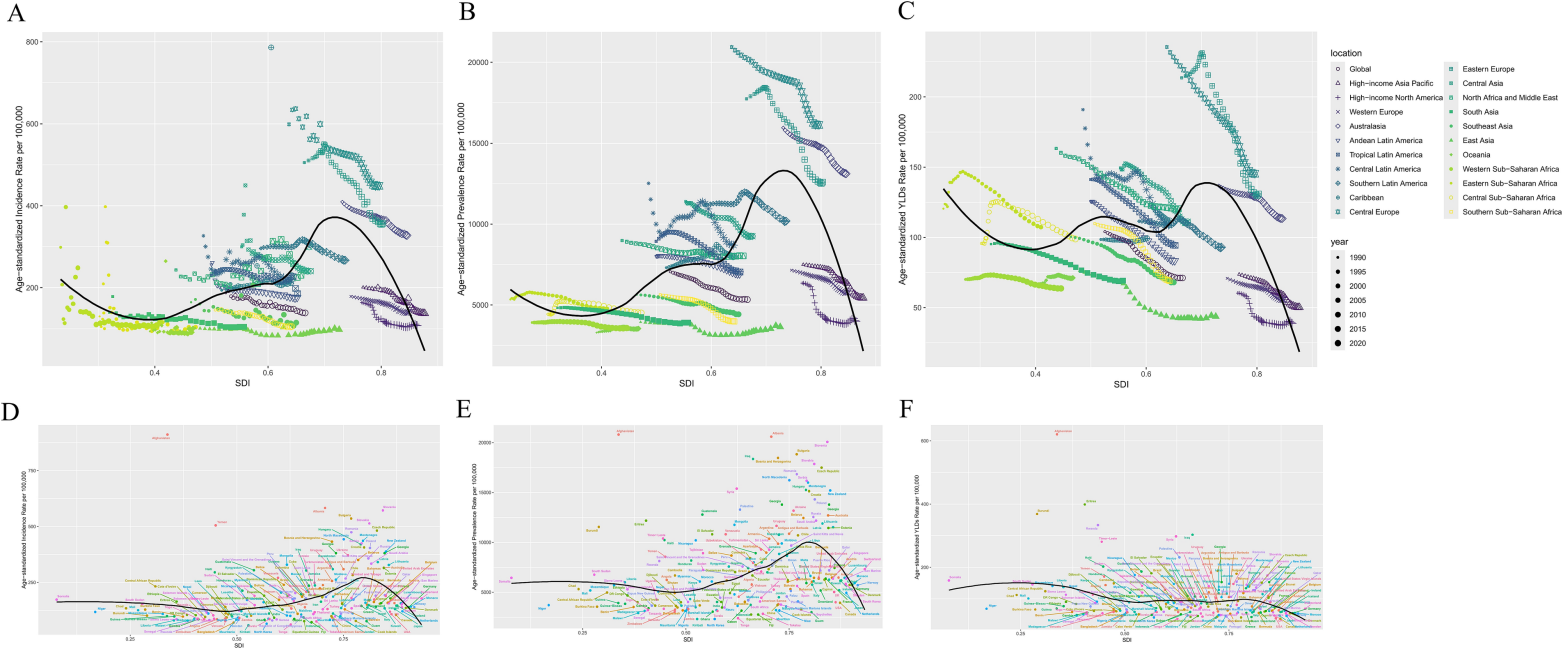
Association of global regional and national burden of traumatic amputations with SDI in 2021. **(A,D)** Age-standardized incidence rates; **(B,E)** Age-standardized prevalence rates; **(C,F)** Age-standardized rates of YLDs.

The age-standardized rate of traumatic amputation burden versus SDI for the 22 GBD regions is shown (Figures 2A–C), with disease burden decreasing at SDI < 0.4 and increasing at SDI > 0.4, with all three measures declining significantly after reaching a peak of 0.7–0.75. All regions showed an overall approximately decreasing trend in burden. Central Europe, Eastern Europe had the greatest decrease in burden over time, but as relatively high SDI regions were much off the curve, with a high amputation burden. The curve of change in burden is flatter in the low SDI and high SDI regions.

In the plot of the burden of traumatic amputation in association with the SDI for 204 countries in 2021, the scatter of incidence and YLDs fits the curve better than that of prevalence. There is also an inflection point in the curve as above, around 0.8, and countries with an SDI above this inflection point have a significantly lower burden of disease. The curves for SDI at 0.25–0.5 and 0.5–0.75 have a V-like shape, where only the 0.5–0.75 part of the YLD is flat. It is worth noting that Afghanistan clearly deviates from the other data points and curves due to high burden (Figures 2D–F).

### 3.4 Gender, age and time trends in the burden of traumatic amputations

The number of prevalence of traumatic amputations in 2021 were 306.6 million (95% UI 281.0–334.8) for males and 138.7 million (125.4–156.5) for females. The number of incidence in 2021 was 7.7 million (6.4–9.1) for males and 3.2 million (2.6–3.9) for females. The number of YLDs with traumatic amputations was 3.9 million (2.6–6.0) years for males and 2.1 million (1.4–3.1) years for females (Supplementary Table 2). Overall, the burden of disease is significantly greater for males than for females. From 1990 to 2021, the burden of disease for traumatic amputation has shown a significant downward trend for both genders (Supplementary Table 2).

**Figure 3:**
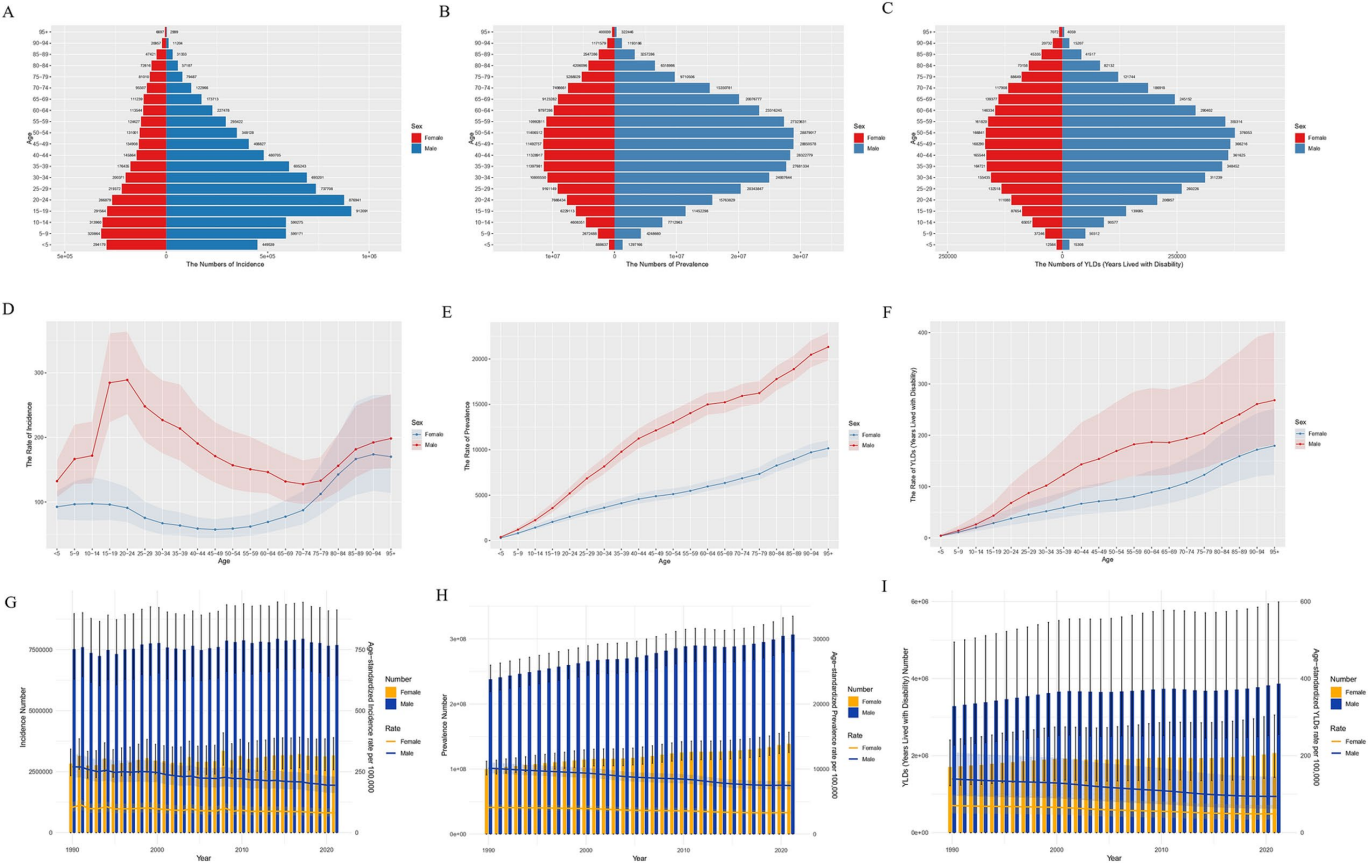
Number of male and female traumatic amputation burden global in 2021. **(A)** Incidence; **(B)** Prevalence; **(C)** YLDs. Rate of male and female traumatic amputation burden global in 2021. **(D)** Incidence; **(E)** Prevalence; **(F)** YLDs. Changes in the number and rate of male and female traumatic amputation burden, 1990–2021. **(G)** Incidence; **(H)** Prevalence; **(I)** YLDs.

The highest number of incidence of traumatic amputations in 2021 was in 15–19 age group (1.2 million [0.9–1.5]) (Supplementary Table 3). It is highest in the 15–19 age group for males and in the 5–9 age group for females, with a decreasing trend in both as age increases (Figure 3A). For the number of prevalence and YLDs, there were similar characteristics, with males and females appearing to have the same peak age ranges of 45–49 and 50–54, and the curves were roughly parabolic (Figures 3B,C). Apparently, the total number for both genders was highest with the number of prevalence in 45–49 age group (40.3 million [36.6–44.5]) and the number of YLDs in 50–54 age group (0.5 million [0.3–0.8] years) (Supplementary Table 3).

For rates of burden, males remain significantly higher than females (Figures 3D–F). Age-standardized incidence peaks in the 15–24 age group for males, and then both genders experience a decline after this age until age 75, when the burden of disease rises significantly again (Figure 3D). The rates of prevalence and YLDs increased with age in both genders (Figures 3E,F).

During 1990–2021, the burden of disease has been significantly greater for males than for females, both in terms of numbers and rates. For numbers, the change of incidence in both genders has tended to be generally stable with little fluctuation (Figure 3G). Both genders showed increasing trends in prevalence and YLDs (Figures 3H,I). For age-standardized rates, both genders showed a decreasing trend in prevalence, incidence, and YLDs over time, with a greater decrease in males than females (Figures 3G–I).

### 3.5 Age-period-cohort (APC) analysis of traumatic amputations

Prevalence and YLDs are more reflective of the accumulation and burden of disease, whereas incidence represents new cases per year, and is more straightforward for analyzing the three elements affecting the model and the model itself. Therefore, we only list the results of the analysis of incidence.

**Figure 4:**
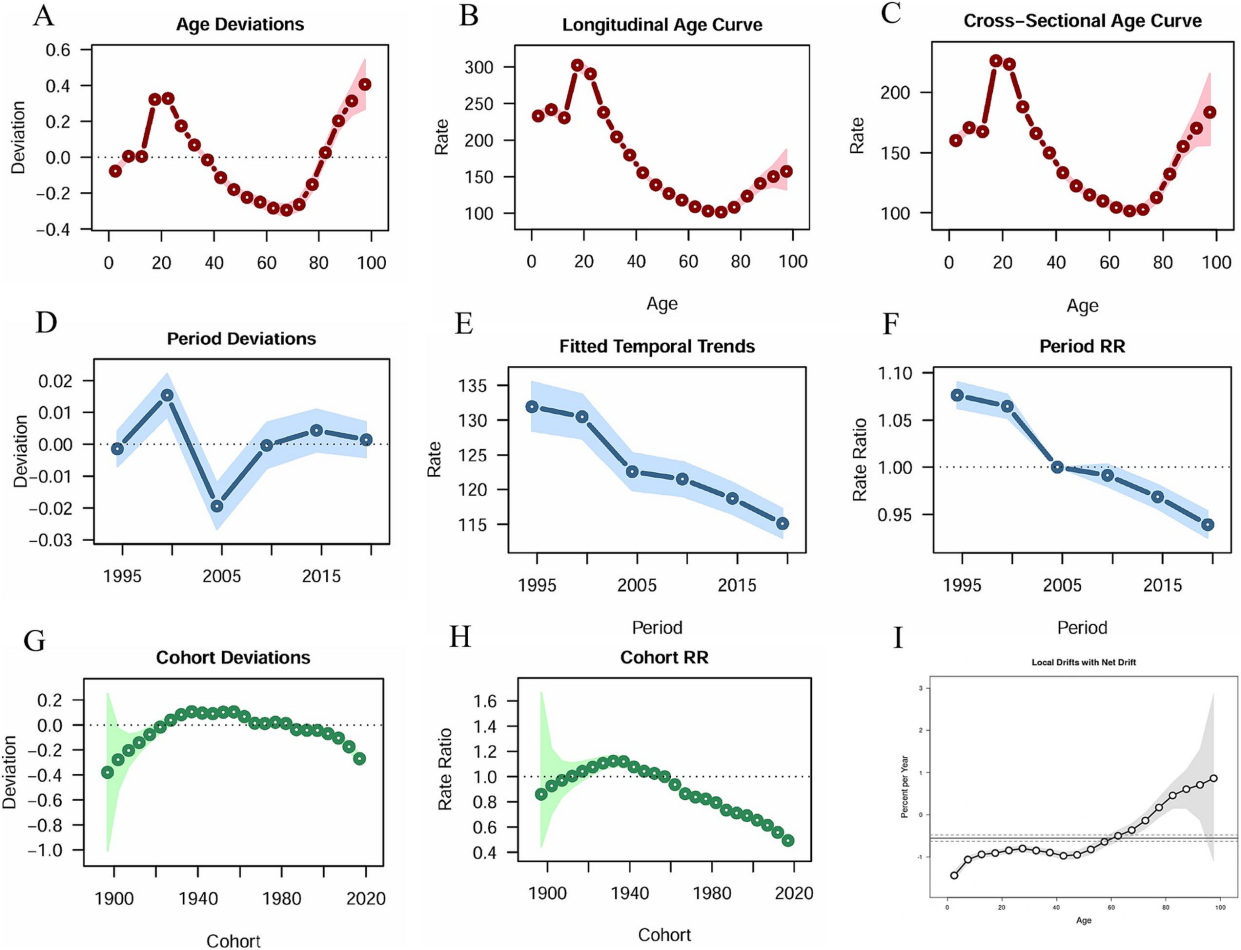
Age-period-cohort analysis results. **(A)** Age Deviations; **(B)** Longitudinal Age Curve; **(C)** Cross-Sectional Age Curve; **(D)** Period Deviations; **(E)** Fitted Temporal Trends; **(F)** Period RR; **(G)** Cohort Deviations; **(H)** Cohort RR; **(I)** Local Drifts with Net Drift.

In terms of age effects, the picture varied by age. The prevalence of amputation showed a fluctuating increase until the age of 20, reached a peak and then began to decline until it reached a minimum around the age of 70 then increased again (Figures 4A–C). Period Deviations were significant for 1997–2001 and 2002–2006 (Figure 4D). For Fitted Temporal Trends, there was an overall downward trend in incidence over time (Figure 4E), here using 2002 to 2006 as the reference group (RR = 1), the RR decreased from 1.076 (95% CI 1.063–1.091) to 0.939 (0.925–0.954) (Figure 4F). Analysis of cohort effects showed less obvious fluctuations in cohort deviation (Figure 4G), with those born after 1957 having a lower incidence of amputation than those born before (RR = 1), with the RR decreasing from 0.86 (95% CI 0.444–1.666) to 0.493 (0.473–0.513), (Figure 4H). From 1990 to 2021, the overall Net Drift (%/year) for incidence was −0.55 (95% CI -0.6 to −0.4), with a general upward trend in the curve and a significant increase after becoming positive after age 77.5 (Figure 4I).

### 3.6 Decomposition analysis of traumatic amputations

**Figure 5:**
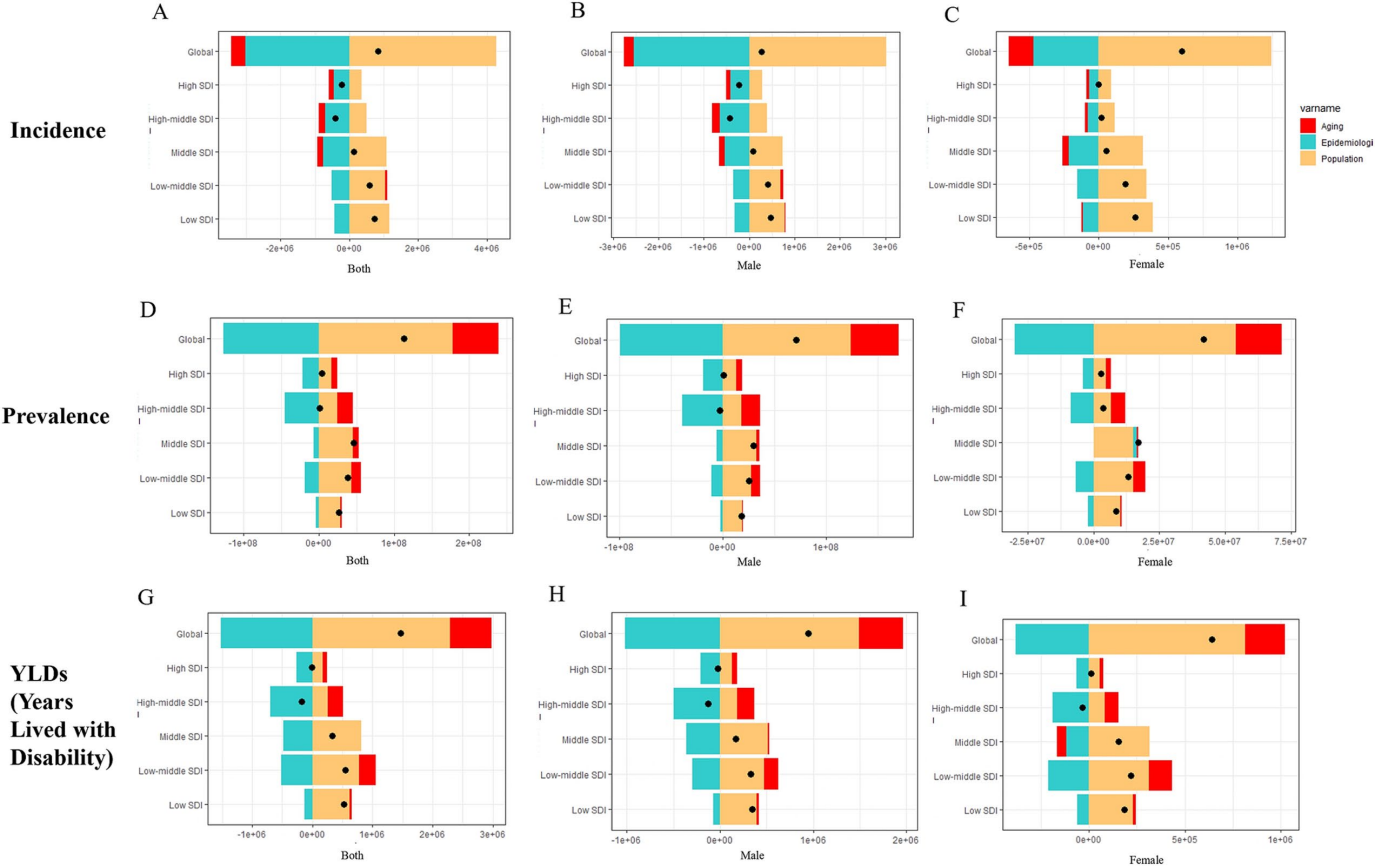
Decomposition analysis results for global and five SDI regions. **(A–C)** Incidence; **(D–F)** Prevalence; **(G–I)** YLDs.

Decomposition analyses revealed the age, epidemiological trends, and population contribution to the burden of disease in the five SDI regions, from high to low, globally in 2021 (Figure 5). In incidence, age was a reducing element of burden in most cases (Figures 5A–C), whereas in prevalence and YLDs it was the exact opposite and accounted for more than the incidence burden (Figures 5D–I). In the vast majority of cases, population increased the burden while the epidemiological trend reduced it (Figure 5). In a regional perspective, SDI and burden tended to be distributed in an “S”-like pattern, but in general regions with high SDI did have relatively low burdens. When analyzed by gender, the pattern of results is similar for males and females in different regions compared to the overall decomposition (Figure 5). For the quantification of the figure above, we present the total burden as well as the quantified values and corresponding percentages for the three elements, and the former is also used for comparison with the difference in the number of burdens. There is general agreement from the results presented, as detailed in Supplementary Table 4.

### 3.7 Prediction of traumatic amputation

**Figure 6:**
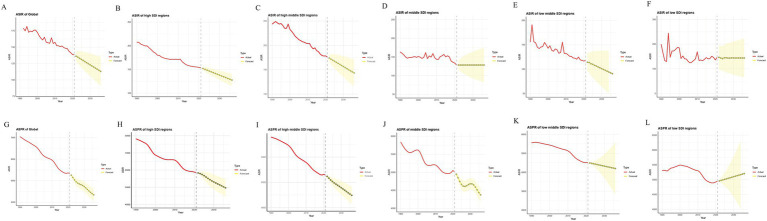
ARIMA projections for the globe and the five SDI regions. **(A–F)** Incidence; **(G–L)** Prevalence. ASIR, Age-standardized incidence rates; ASPR, Age-standardized prevalence rates.

Since the YLDs were calculated using the disability weighting parameters, the confidence in their predicted up results was relatively weak, so we chose to do a 15-year prediction of incidence and prevalence rates using ARIMA (Figures 6A–L). The derived different fitted models (p, d, q) and other parameters using the auto.arima() function are presented in detail in Supplementary Table 5. Except for the incidence in the low SDI region, the rest of the observations are in good agreement with the fitted values. Regarding residence analysis, incidence and prevalence in low SDI region, incidence in low-middle SDI region did not pass the PACF plot test. Incidence in low SDI region, prevalence in high SDI region did not pass the ACF plot test. All data passed the QQ plot test. The above illustrates that there are individual instances in the forecast where the data does not conform to a normal distribution. (Due to space constraints, three plots were not placed in the results as a judgment tool only). All models passed the Ljung-Box test.

Based on these models, we predicted that over the next 15 years, global incidence would fall from 138.5 per 100,000 in 2021 to 113.0 per 100,000 in 2036, and prevalence would fall from 5333.4 per 100,000 in 2021 to 4339.9 per 100,000 in 2036. Higher SDI regions would have significantly lower incidence and prevalence rates (Figures 6B,C,H,I). The rates of incidence and prevalence in the middle and low-middle SDI regions will decrease or remain stable, and the low SDI regions will face higher prevalence rates (Figures 6D–F,J,K).

### 3.8 Etiologic analysis of traumatic amputations

**Figure 7:**
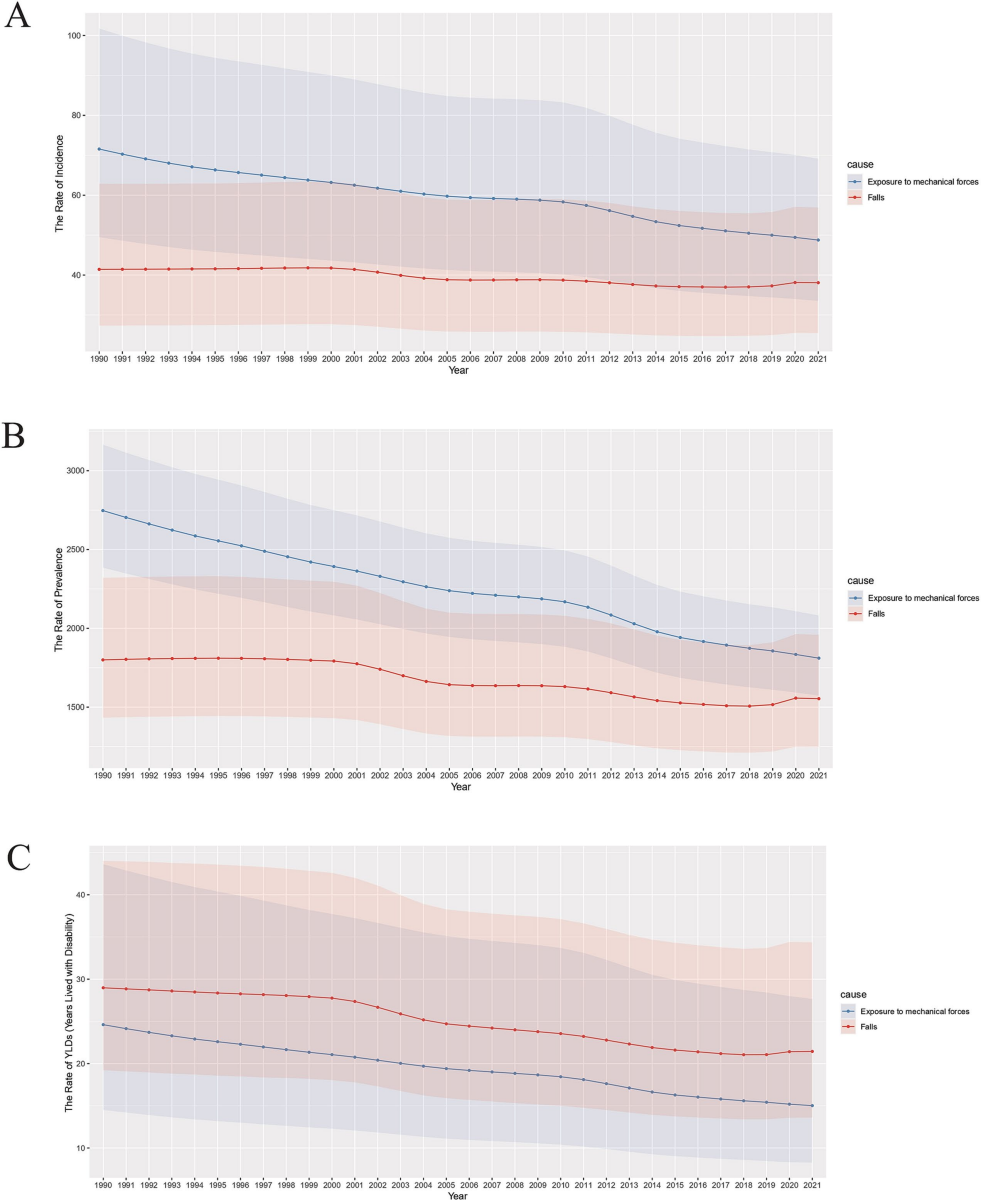
Burden of exposure to mechanical forces and falls for both genders, 1990–2021. **(A)** Age-standardized incidence rates; **(B)** Age-standardized prevalence rates; **(C)** Age-standardized YLDs rates.

In 2021, the highest burden of disease for traumatic amputations was due to exposure to mechanical forces and falls and was much higher than other causes (Supplementary Table 6). We performed a curve analysis of time and age-standardized rates for both causes of injury. With the exception of falls in which the curve for incidence tended to flatten out (Figure 7A), the rest of the curves showed a downward trend with occasional fluctuations over time (Figures 7A–C). Notably, the curves of incidence and prevalence of exposure to mechanical forces were both higher than falls, while the opposite was true for YLDs (Figures 7A–C).

## 4 Discussion

The global incidence number of trauma-related amputations increased by only about 5% from 1990 to 2021, showing mainly numerical fluctuations during this period. However, the number of prevalence and YLDs increased significantly. In contrast, age-standardized rates showed a significant downward trend. Over the past 30 years, the world has experienced population growth and aging (15), with the accumulation of cases implying an increase in prevalence, and the prolonged duration of disability in the older adult has led to a significant increase in the number of prevalence and YLDs. Advances in medical technology have led to more scientific management of amputations in some cases (16) and developments in care and rehabilitation have reduced disability (17, 18) and lowered the age-standardized rate of disease burden. Age-standardized rate measures contributed to this trend by neutralizing population changes.

India and China have large populations with high numbers of amputation burdens, while the opposite is found in some countries in the Western Pacific. Age-standardized rates of amputation burden in 2021 were particularly high in Central and Eastern Europe, the Middle East, the Caribbean, New Zealand, and Australia (Figure 1). Combined with the change from 1990 to 2021 (Table 1) and the association between SDI and amputation burden in different regions and countries (Figure 2), the rising 0.4–0.7 SDI burden curve might be associated with rapid industrial development and road transportation pressures accompanying GDP growth (19, 20).

There are differences in SDI between regions and countries with the highest and lowest burdens, but SDI is not the sole determinant of amputation burden; region-specific policies or events also play a role. Central and Eastern Europe and the Middle East are plagued by conflict and regional instability (21–23). Significant regional inequalities in burden exist in Europe, and since 1990, Central and Eastern European countries have experienced economic transition accompanied by an increase in violence and unintentional injuries, and the existence of an under-appreciated burden of disease from injuries (24–26). Trauma patterns and systems in New Zealand and Australia are similar, with low levels of rural health care, poor transportation design and quality, and geographic issues that often lead to delays in treatment (27, 28). Inadequate urban planning, unsafe neighborhoods and inadequate health care exist in the Caribbean and Latin American regions (29). In addition, it is hypothesized here that the mechanisms for monitoring and reporting epidemiology are better in more developed countries compared to less developed countries. Moreover, there are differences in data collection methods, case definition methods, and so on, in different countries. These may lead to differences in data quality, which in turn may affect the judgment of the relationship between SDI and the burden of traumatic amputations (30).

The burden of traumatic amputation was much heavier for males than for females in the period 1990–2021. This may be due to the fact that males are exposed to more risks on a daily basis, such as strenuous physical labor, traffic accidents, violence, etc. (9, 31, 32). This explains the peak rate of incidence in males aged 15–24 years and the high rate of incidence in young adults (Figures 3D, 4B). The burden for both genders rose significantly and converged at age 75 (Figure 3D), driven by a combination of declining body function and the same external environment. The burden of age-standardized rates declined slightly more for males than for females (Figures 3G–I), which we hypothesize is because of improved economic conditions, population health driven by a steady increase in global SDI over this time period (33), manifested in terms of amputation burden may imply increased labor security, facility safety, and safety awareness. Moreover, the results in Figure 4E are consistent with these observations. The number of prevalence and YLDs is concentrated in young adults, which should be of great concern to health care policy. The high age-standardized rates in the older age groups stem from the cumulative effect of the 1912–1957 birth cohort, a period when the world was at war and medical care was far less developed than today (Figure 4H).

According to Das Gupta’s methodology, (12, 13) the analysis (Figure 5) shows that the patterns of males and females in different regions are basically the same. Changing age structure reduced the burden of incidence but increased prevalence and YLDs, probably due to the combined effect of aging and oligarchic trends (15) in most countries worldwide to reduce the absolute number of amputation incidence, especially in high SDI regions. Older people have greater prevalence of and YLDs than younger people (Figure 3F), allowing changes in age structure to increase prevalence of and YLDs. Increasing population implies increasing burdens, which are more pronounced in lower SDI countries, and the cumulative effect is exacerbated by an increase in the average age of the population (15). Encouragingly, epidemiologic reductions were observed in the vast majority of subgroups, suggesting that global measures to address the burden associated with amputation have been successful, and the projections are consistent with this. However, the high fluctuation in burden in the lower SDI regions limits the reliability of the projections, and some of the residuals do not conform to a normal distribution, but the short-term projections are still informative.

Finger amputation accounts for the largest proportion of traumatic amputations, and in the United States, for example, the cause are often mechanical forces (32). Our results show that the prevalence and incidence of mechanical forces causes are higher than falls, but the opposite is true for YLDs, which implies that falls are more injurious, but less likely to occur. Falls are an important cause of death and disability worldwide, and can even be accompanied by brain, spinal cord, and visceral injuries, which are extremely harmful, yet have not attracted a great deal of attention from healthcare and social policy makers (34). Therefore, the age-standardized prevalence of falls has consistently leveled off (Figure 7A).

In summary, the major risks of traumatic amputation vary by region and age, and health interventions tailored to different populations should be customized to reduce the burden of disease, rather than as a one-size-fits-all package. The limitations of this study are (35), firstly, at present, the latest data in the GBD database is only updated to 2021, which has a certain lag. We chose to use 1990 as the starting year, which is the usual practice in GBD research, because the previous data are indeed somewhat outdated when compared and analyzed with the 2021 data, but it does not mean that they are not meaningful for studies. In addition, there may be bias in the data from less developed regions. Secondly, YLD comparisons need to take into account local levels of social development. Finally, different amputations have different characteristics and burdens, and differences in the proportion of the overall population can interfere with the understanding of the results. Nonetheless, our analyses can provide important information to inform the development of strategies to reduce the burden of traumatic amputations.

## 5 Conclusion

The global burden of traumatic amputations increased in number from 1990 to 2021, but the age-standardized rate of burden declined significantly and is expected to continue to decline in the future. Population growth is now the main cause of the burden, and more attention needs to be paid to men, youth and the older adult.

## Data Availability

The raw data supporting the conclusions of this article will be made available by the authors, without undue reservation.

## Footnotes

^1^ http://ghdx.healthdata.org/gbd-results-tool
