# Supplementary material for: Global, regional and national burden of traumatic amputations from 1990 to 2021: a systematic analysis of the Global Burden of Disease study 2021
Source: Front Public Health. 2025 Jun 2;13:1583523. doi: 10.3389/fpubh.2025.1583523 (PMC12171122; doi:10.3389/fpubh.2025.1583523)
Supplement: Supplementary file 1 [file Table_1.docx]

Supplementary table 1: Age-standardized rates of incidence, prevalence, YLDs by region, 2021.

|  | Incidence | Prevalence | Years lived with disability (YLDs) |
| --- | --- | --- | --- |
|  | 2021 (per 100000) | 2021 (per 100000) | 2021 (per 100000) |
| Global | 139(116,165) | 5333(4895,5818) | 71(48,109) |
| East Asia | 98(79,121) | 3645(3291,4100) | 44(27,70) |
| Southeast Asia | 115(97,135) | 4424(4026,4962) | 68(48,101) |
| Oceania | 101(87,121) | 3542(3224,3921) | 71(52,95) |
| Central Asia | 241(194,300) | 9252(8559,10132) | 111(73,174) |
| Central Europe | 448(343,576) | 16129(14704,17803) | 145(79,263) |
| Eastern Europe | 357(286,436) | 12592(11607,13834) | 131(78,219) |
| High-income Asia Pacific | 137(104,179) | 5435(4966,6022) | 50(26,89) |
| Australasia | 328(241,436) | 13110(11638,14827) | 113(56,213) |
| Western Europe | 139(107,179) | 5760(5139,6600) | 56(30,99) |
| Southern Latin America | 267(197,358) | 10187(9182,11382) | 93(51,167) |
| High-income North America | 107(85,135) | 3990(3699,4345) | 39(21,67) |
| Caribbean | 230(189,279) | 8027(7400,8741) | 112(77,167) |
| Andean Latin America | 176(142,217) | 6923(6369,7568) | 83(55,131) |
| Central Latin America | 216(173,270) | 8699(7897,9657) | 109(72,167) |
| Tropical Latin America | 186(153,226) | 7039(6370,7850) | 94(64,141) |
| North Africa and Middle East | 230(191,278) | 8035(7059,9380) | 120(79,185) |
| South Asia | 104(87,124) | 3901(3545,4345) | 69(50,96) |
| Central Sub-Saharan Africa | 104(89,121) | 4518(3837,5499) | 99(68,143) |
| Eastern Sub-Saharan Africa | 116(95,141) | 4775(4058,5926) | 107(72,163) |
| Southern Sub-Saharan Africa | 106(89,124) | 3976(3698,4303) | 69(50,96) |
| Western Sub-Saharan Africa | 99(84,117) | 3572(3279,3905) | 64(47,87) |
