# Supplementary material for: Global, regional and national burden of traumatic amputations from 1990 to 2021: a systematic analysis of the Global Burden of Disease study 2021
Source: Front Public Health. 2025 Jun 2;13:1583523. doi: 10.3389/fpubh.2025.1583523 (PMC12171122; doi:10.3389/fpubh.2025.1583523)
Supplement: Supplementary file 2 [file Table_2.docx]

Supplementary table 2: Gender comparisons of the global burden of traumatic amputations (incidence, prevalence, YLDs) in 1990 and 2021 in terms of numbers and age-standardized rates of change

|  | | 1990(thousands) | 2021(thousands) | 1990-2021 age-standardized rate change (per 100000) |
| --- | --- | --- | --- | --- |
| Incidence | Male | 7522(6290,8979) | 7691(6441,9130) | -0.28(-0.3,-0.26) |
|  | Female | 2831(2331,3427) | 3169(2581,3896) | -0.22(-0.24,-0.19) |
| Prevalence | Male | 237935(218900,259599) | 306560(281031,334836) | -0.26(-0.28,-0.25) |
|  | Female | 100164(90952,111879) | 138678(125390,156494) | -0.2(-0.22,-0.17) |
| YLDS（Years lived with disability） | Male | 3284(2274,4948) | 3868(2550,5991) | -0.33(-0.37,-0.29) |
|  | Female | 1704(1219,2406) | 2067(1434,3052) | -0.3(-0.36,-0.25) |
