# Supplementary material for: Global, regional and national burden of traumatic amputations from 1990 to 2021: a systematic analysis of the Global Burden of Disease study 2021
Source: Front Public Health. 2025 Jun 2;13:1583523. doi: 10.3389/fpubh.2025.1583523 (PMC12171122; doi:10.3389/fpubh.2025.1583523)
Supplement: Supplementary file 3 [file Table_3.docx]

Supplementary table 3: Number of incidence, prevalence, YLDS, by age group, 2021.

|  | Incidence | Prevalence | YLDS（Years lived with disability） |
| --- | --- | --- | --- |
|  | 2021 (thousands) | 2021 (thousands) | 2021 (thousands) |
| <5 years | 744(602,940) | 2186(1948,2433) | 28(19,43) |
| 5-9 years | 911(698,1209) | 6921(5796,8450) | 88(56,142) |
| 10-14 years | 904(670,1191) | 12321(10505,14855) | 156(99,243) |
| 15-19 years | 1204(943,1534) | 17681(15406,20632) | 227(147,353) |
| 20-24 years | 1144(924,1461) | 23430(20607,26639) | 318(210,491) |
| 25-29 years | 957(749,1207) | 29505(26336,33149) | 393(261,599) |
| 30-34 years | 894(688,1147) | 35738(32055,40087) | 467(309,714) |
| 35-39 years | 782(603,1026) | 39079(35248,43162) | 513(341,790) |
| 40-44 years | 627(478,808) | 39652(35887,43705) | 527(351,807) |
| 45-49 years | 542(417,693) | 40343(36638,44488) | 535(355,821) |
| 50-54 years | 479(369,624) | 40286(36773,44409) | 543(361,837) |
| 55-59 years | 418(322,550) | 38316(34936,41905) | 517(344,797) |
| 60-64 years | 341(267,435) | 33114(30624,35881) | 437(289,671) |
| 65-69 years | 285(221,374) | 29200(27037,31569) | 385(258,582) |
| 70-74 years | 218(166,281) | 22849(21193,24772) | 305(208,458) |
| 75-79 years | 160(120,214) | 14999(13864,16266) | 210(146,313) |
| 80-84 years | 130(95,180) | 10725(9947,11684) | 155(107,226) |
| 85-89 years | 79(56,115) | 5805(5387,6327) | 87(60,126) |
| 90-94 years | 32(23,47) | 2365(2199,2553) | 36(25,52) |
| 95+ years | 10(7,14) | 722(668,775) | 11(8,16) |
