# Supplementary material for: Global, regional and national burden of traumatic amputations from 1990 to 2021: a systematic analysis of the Global Burden of Disease study 2021
Source: Front Public Health. 2025 Jun 2;13:1583523. doi: 10.3389/fpubh.2025.1583523 (PMC12171122; doi:10.3389/fpubh.2025.1583523)
Supplement: Supplementary file 4 [file Table_4.docx]

Supplementary table 4: Values from decomposition analysis results for global and 5 SDI regions.

|  | location_name | overll_difference | a_effect | p_effect | r_effect | a_percent | p_percent | r_percent | val_1990 | val_2021 | diff1 |
| --- | --- | --- | --- | --- | --- | --- | --- | --- | --- | --- | --- |
| Both | Global | 841440.34 | -405756.391 | 4277522.379 | -3030325.652 | -48.22 | 508.36 | -360.14 | 10352277 | 10859433 | 507156.1 |
|  | High SDI | -227527.8 | -131824.703 | 365247.381 | -460950.477 | 57.94 | -160.53 | 202.59 | 1774416 | 1546888 | -227527.8 |
|  | High-middle SDI | -406159.29 | -200770.948 | 493207.402 | -698595.747 | 49.43 | -121.43 | 172 | 2635262 | 2204443 | -430819.3 |
|  | Middle SDI | 134740.62 | -177042.071 | 1074182.855 | -762400.163 | -131.39 | 797.22 | -565.83 | 2967183 | 3101923 | 134740.6 |
|  | Low-middle SDI | 588087.96 | 57965.214 | 1039923.705 | -509800.961 | 9.86 | 176.83 | -86.69 | 1877357 | 2273026 | 395669.2 |
|  | Low SDI | 727648.9 | 1447.834 | 1165741.194 | -439540.123 | 0.2 | 160.21 | -60.41 | 1080894 | 1718570 | 637675.6 |
| Male | Global | 264486.35 | -215066.675 | 3019674.672 | -2540121.645 | -81.31 | 1141.71 | -960.4 | 7521753.3 | 7690618 | 168864.3 |
|  | High SDI | -224782.29 | -103987.101 | 287101.034 | -407896.221 | 46.26 | -127.72 | 181.46 | 1344634 | 1119852 | -224782.3 |
|  | High-middle SDI | -422066.37 | -159204.562 | 389808.023 | -652669.83 | 37.72 | -92.36 | 154.64 | 2042963.8 | 1636378 | -406585.9 |
|  | Middle SDI | 79240.3 | -118123.779 | 742486.855 | -545122.773 | -149.07 | 937.01 | -687.94 | 2123416.9 | 2202657 | 79240.3 |
|  | Low-middle SDI | 403684.27 | 67323.045 | 687573.152 | -351211.928 | 16.68 | 170.32 | -87 | 1267119.4 | 1535963 | 268844 |
|  | Low SDI | 473485.99 | 14318.792 | 776290.027 | -317122.83 | 3.02 | 163.95 | -66.98 | 730040.6 | 1184649 | 454608.3 |
| Female | Global | 598090.39 | -177967.604 | 1245497.916 | -469439.917 | -29.76 | 208.25 | -78.49 | 2830523.9 | 3168815.7 | 338291.872 |
|  | High SDI | -2745.51 | -23477.706 | 88520.214 | -67788.02 | 855.13 | -3224.18 | 2469.05 | 429781.8 | 427036.2 | -2745.511 |
|  | High-middle SDI | 15850.92 | -20986.051 | 113038.874 | -76201.907 | -132.4 | 713.14 | -480.74 | 592298.2 | 568064.8 | -24233.374 |
|  | Middle SDI | 55500.32 | -46809.271 | 317381.576 | -215071.987 | -84.34 | 571.86 | -387.51 | 843766 | 899266.3 | 55500.318 |
|  | Low-middle SDI | 189997.49 | -549.974 | 345415.846 | -154868.383 | -0.29 | 181.8 | -81.51 | 610237.3 | 737062.5 | 126825.201 |
|  | Low SDI | 261713.78 | -11639.923 | 388587.317 | -115233.616 | -4.45 | 148.48 | -44.03 | 350853.3 | 533920.7 | 183067.335 |
| Both | Global | 112733908 | 61264435.46 | 177606346.3 | -126136873.7 | 54.34 | 157.54 | -111.89 | 338098873 | 445237917 | 107139044 |
|  | High SDI | 3839120.73 | 8122502.499 | 17066179.77 | -21349561.53 | 211.57 | 444.53 | -556.11 | 76481638 | 81839283 | 5357646 |
|  | High-middle SDI | 921557.72 | 21135268.44 | 24471071.46 | -44684782.18 | 2293.43 | 2655.4 | -4848.83 | 102641627 | 106626537 | 3984910 |
|  | Middle SDI | 46683442.75 | 7064363.411 | 45896617.57 | -6277538.233 | 15.13 | 98.31 | -13.45 | 87235902 | 134009176 | 46773274 |
|  | Low-middle SDI | 38536423.2 | 13392820.88 | 43108438.85 | -17964836.52 | 34.75 | 111.86 | -46.62 | 51003804 | 79816802 | 28812998 |
|  | Low SDI | 26670030.97 | 1671153.977 | 29023816.39 | -4024939.389 | 6.27 | 108.83 | -15.09 | 20111899 | 42306340 | 22194441 |
| Male | Global | 71456873.37 | 46464007.57 | 124010942 | -99018076.24 | 65.02 | 173.55 | -138.57 | 237934629 | 306559959 | 68625329.7 |
|  | High SDI | 590045.83 | 6214973.369 | 13073601.9 | -18698529.44 | 1053.3 | 2215.69 | -3169 | 54924210 | 57123276 | 2199066.6 |
|  | High-middle SDI | -2820343.38 | 17557693.55 | 18561465.24 | -38939502.17 | -622.54 | -658.13 | 1380.67 | 75549617 | 75606961 | 57344.6 |
|  | Middle SDI | 30137654.27 | 3084092.577 | 32699241.35 | -5645679.654 | 10.23 | 108.5 | -18.73 | 60753111 | 93154252 | 32401140.8 |
|  | Low-middle SDI | 25796805.27 | 8628660.507 | 27688974.42 | -10520829.66 | 33.45 | 107.33 | -40.78 | 33256178 | 52274507 | 19018329.7 |
|  | Low SDI | 18048514.9 | 1060242.613 | 18872547.71 | -1884275.421 | 5.87 | 104.57 | -10.44 | 12975637 | 27929966 | 14954329.5 |
| Female | Global | 41706492.47 | 17376831.97 | 54050899.77 | -29721239.27 | 41.66 | 129.6 | -71.26 | 100164244 | 138677958 | 38513714 |
|  | High SDI | 2750807.61 | 1764750.543 | 4842720.151 | -3856663.081 | 64.15 | 176.05 | -140.2 | 21557428 | 24716007 | 3158579 |
|  | High-middle SDI | 3614279.6 | 5655922.2 | 6552549.719 | -8594192.322 | 156.49 | 181.3 | -237.78 | 27092010 | 31019576 | 3927566 |
|  | Middle SDI | 17128722.22 | 719260.531 | 15042919.87 | 1366541.823 | 4.2 | 87.82 | 7.98 | 26482790 | 40854924 | 14372134 |
|  | Low-middle SDI | 13189719.51 | 4666043.879 | 15184550.87 | -6660875.232 | 35.38 | 115.12 | -50.5 | 17747627 | 27542295 | 9794668 |
|  | Low SDI | 8658916.87 | 569602.149 | 10110209.24 | -2020894.518 | 6.58 | 116.76 | -23.34 | 7136263 | 14376374 | 7240111 |
| Both | Global | 1467176.32 | 693151.16 | 2288964.876 | -1514939.717 | 47.24 | 156.01 | -103.26 | 4988017.4 | 5935830.8 | 947813.474 |
|  | High SDI | -8115.5 | 79815.529 | 171847.543 | -259778.572 | -983.49 | -2117.52 | 3201.02 | 784483.1 | 787769.8 | 3286.738 |
|  | High-middle SDI | -176654.86 | 250605.191 | 264842.978 | -692103.027 | -141.86 | -149.92 | 391.78 | 1327224.5 | 1135358 | -191866.469 |
|  | Middle SDI | 338253.23 | -442.116 | 817214.1 | -478518.751 | -0.13 | 241.6 | -141.47 | 1499534.5 | 1830316.5 | 330781.968 |
|  | Low-middle SDI | 544686.09 | 278131.474 | 784885.04 | -518330.427 | 51.06 | 144.1 | -95.16 | 932295 | 1306458.7 | 374163.697 |
|  | Low SDI | 528184.31 | 38948.783 | 621005.518 | -131769.992 | 7.37 | 117.57 | -24.95 | 436880.2 | 868545.8 | 431665.566 |
| Male | Global | 947260.01 | 473302.079 | 1491232.271 | -1017274.341 | 49.97 | 157.43 | -107.39 | 3284368.6 | 3868495.1 | 584126.54 |
|  | High SDI | -22032.83 | 60219.537 | 123573.631 | -205825.998 | -273.32 | -560.86 | 934.18 | 534517.6 | 523881.4 | -10636.26 |
|  | High-middle SDI | -127310.7 | 183099.766 | 186378.004 | -496788.47 | -143.82 | -146.4 | 390.22 | 907718.4 | 764438.8 | -143279.6 |
|  | Middle SDI | 163911.58 | 14642.918 | 514275.75 | -365007.091 | 8.93 | 313.75 | -222.69 | 987582.8 | 1213675.3 | 226092.45 |
|  | Low-middle SDI | 329721.34 | 158254.839 | 469133.985 | -297667.488 | 48 | 142.28 | -90.28 | 574782.2 | 804525.3 | 229743.03 |
|  | Low SDI | 345192.24 | 24386.913 | 391700.261 | -70894.933 | 7.06 | 113.47 | -20.54 | 274328.8 | 556962.2 | 282633.41 |
| Female | Global | 641273.47 | 206150.048 | 814864.971 | -379741.545 | 32.15 | 127.07 | -59.22 | 1703648.8 | 2067335.7 | 363686.93 |
|  | High SDI | 9922.34 | 19799.265 | 54515.196 | -64392.121 | 199.54 | 549.42 | -648.96 | 249965.5 | 263888.5 | 13922.99 |
|  | High-middle SDI | -33465.17 | 71944.154 | 83621.646 | -189030.97 | -214.98 | -249.88 | 564.86 | 419506.1 | 370919.2 | -48586.87 |
|  | Middle SDI | 152721.55 | -46299.969 | 317367.064 | -118345.542 | -30.32 | 207.81 | -77.49 | 511951.7 | 616641.2 | 104689.52 |
|  | Low-middle SDI | 219035.96 | 118988.092 | 312466.984 | -212419.113 | 54.32 | 142.66 | -96.98 | 357512.8 | 501933.5 | 144420.66 |
|  | Low SDI | 182872.99 | 14163.271 | 228803.695 | -60093.977 | 7.74 | 125.12 | -32.86 | 162551.4 | 311583.6 | 149032.16 |
