# Supplementary material for: Global, regional and national burden of traumatic amputations from 1990 to 2021: a systematic analysis of the Global Burden of Disease study 2021
Source: Front Public Health. 2025 Jun 2;13:1583523. doi: 10.3389/fpubh.2025.1583523 (PMC12171122; doi:10.3389/fpubh.2025.1583523)
Supplement: Supplementary file 5 [file Table_5.docx]

Supplementary table 5: ARIMA's parameters.

|  |  |  | Observation and fitting values | | | | | Ljung-Box | |
| --- | --- | --- | --- | --- | --- | --- | --- | --- | --- |
| Measure name | location | ARIMA model | Cor | p | AIC | AICc | BIC | Ljung-Box χ2 | Ljung-Box p |
| Incidence | Global | (1,1,0) | 0.96 | <2.20E-16 | 179.52 | 180.40 | 183.82 | 0.25 | 0.62 |
|  | High SDI | (0,1,0) | 0.99 | <2.20E-16 | 123.25 | 123.68 | 126.12 | 2.21 | 0.14 |
|  | High-middle SDI | (0,1,0) | 0.99 | <2.20E-16 | 170.67 | 171.10 | 173.54 | 0.78 | 0.38 |
|  | Middle SDI | (0,1,0) | 0.67 | 2.91E-05 | 202.13 | 202.27 | 203.56 | 3.58 | 0.06 |
|  | Low-middle SDI | (1,1,0) | 0.82 | 1.03E-08 | 233.00 | 233.89 | 237.31 | 0.06 | 0.81 |
|  | Low SDI | (3,1,0) | 0.50 | 0.003 | 293.33 | 294.87 | 299.07 | 0.06 | 0.80 |
| Prevalence | Global | (2,1,0) | 0.99 | <2.20E-16 | 262.53 | 264.06 | 268.26 | 0.06 | 0.81 |
|  | High SDI | (2,1,0) | 0.99 | <2.20E-16 | 272.88 | 274.41 | 278.61 | 2.13 | 0.14 |
|  | High-middle SDI | (2,1,0) | 0.99 | <2.20E-16 | 309.67 | 311.21 | 315.41 | 0.03 | 0.85 |
|  | Middle SDI | (2,1,1) | 0.99 | <2.20E-16 | 279.76 | 282.16 | 286.93 | 0.34 | 0.56 |
|  | Low-middle SDI | (1,2,0) | 0.99 | <2.20E-16 | 239.43 | 239.87 | 242.23 | 0.08 | 0.78 |
|  | Low SDI | (0,2,0) | 0.99 | <2.20E-16 | 268.13 | 268.27 | 269.53 | 1.36 | 0.24 |
| Note: The value of 0.99 in the Cor column is written as 1 without rounding the original value to the third decimal place. | | | | | | | | | |
