# Supplementary material for: Global, regional and national burden of traumatic amputations from 1990 to 2021: a systematic analysis of the Global Burden of Disease study 2021
Source: Front Public Health. 2025 Jun 2;13:1583523. doi: 10.3389/fpubh.2025.1583523 (PMC12171122; doi:10.3389/fpubh.2025.1583523)
Supplement: Supplementary file 6 [file Table_6.docx]

Supplementary table 6: Number and age-standardized rates of the burden of traumatic amputations of different causes (incidence, prevalence, YLDs) in 2021

| Causes of injury | Incidence | | Prevalence | | YLDS（Years lived with disability） | |
| --- | --- | --- | --- | --- | --- | --- |
|  | 2021 (thousands) | 2021 age-standardized rate (per 100000) | 2021 (thousands) | 2021 age-standardized rate (per 100000) | 2021 (thousands) | 2021 age-standardized rate (per 100000) |
| All causes | 10859(9064,12882) | 138.5(115.6,164.8) | 445238(408968,485841) | 5333.4(4894.6,5818.4) | 5936(3988,9067) | 71.1(47.8,108.6) |
| Transport injuries | 766(575,1033) | 9.6(7.2,13) | 30261(27789,33117) | 359.5(330,393.9) | 681(477,946) | 8.1(5.7,11.2) |
| Road injuries | 669(485,924) | 8.4(6,11.6) | 25861(23443,28165) | 306.7(278.2,333.9) | 544(380,764) | 6.4(4.5,9) |
| Other transport injuries | 96(64,138) | 1.2(0.8,1.8) | 4400(3536,5831) | 52.8(42.4,70) | 136(94,198) | 1.6(1.1,2.4) |
| Unintentional injuries | 8645(6876,10648) | 110(87.4,135.6) | 359150(328026,398564) | 4299.6(3930.8,4774.8) | 3959(2527,6343) | 47.4(30.2,76.1) |
| Falls | 3008(2019,4450) | 38.1(25.5,56.9) | 129930(104582,163244) | 1553.7(1247.5,1959.5) | 1795(1143,2875) | 21.4(13.6,34.4) |
| Drowning | 18(11,29) | 0.2(0.1,0.4) | 695(580,832) | 8.4(7,10) | 13(9,19) | 0.2(0.1,0.2) |
| Fire, heat, and hot substances | 98(58,160) | 1.3(0.8,2.1) | 4210(3445,5369) | 50.5(41.3,64.7) | 53(34,82) | 0.6(0.4,1) |
| Poisonings | 14(9,21) | 0.2(0.1,0.3) | 746(620,934) | 9(7.5,11.2) | 17(11,24) | 0.2(0.1,0.3) |
| Exposure to mechanical forces | 3863(2658,5485) | 48.8(33.5,69.1) | 151935(132138,174304) | 1810.8(1573,2081.9) | 1259(693,2317) | 15(8.3,27.7) |
| Adverse effects of medical treatment | 0(0,0) | 0(0,0) | 0(0,0) | 0(0,0) | 0(0,0) | 0(0,0) |
| Animal contact | 487(288,797) | 6.3(3.7,10.3) | 18539(15963,21826) | 223.2(192,263.1) | 211(137,323) | 2.5(1.6,3.9) |
| Foreign body | 284(160,463) | 3.9(2.2,6.4) | 14251(11169,18518) | 175.6(137.1,228.8) | 181(110,294) | 2.2(1.3,3.6) |
| Exposure to forces of nature | 29(16,51) | 0.4(0.2,0.7) | 3893(2137,6666) | 48.6(26.7,83.5) | 76(39,139) | 1(0.5,1.7) |
| Environmental heat and cold exposure | 84(54,124) | 1.1(0.7,1.6) | 3793(3208,4532) | 45.8(38.7,54.8) | 58(38,86) | 0.7(0.5,1) |
| Self-harm and interpersonal violence | 1449(1031,2003) | 18.9(13.5,26.3) | 55829(42446,75886) | 674.3(512.3,917.3) | 1296(823,2004) | 15.6(9.9,24.2) |
| Self-harm | 101(65,147) | 1.2(0.8,1.8) | 3567(2948,4335) | 42(34.7,51.2) | 137(95,186) | 1.6(1.1,2.2) |
| Interpersonal violence | 481(307,718) | 6.2(4,9.3) | 20139(17803,23004) | 242.5(214.6,277.2) | 360(252,513) | 4.3(3,6.2) |
| Conflict and terrorism | 707(399,1190) | 9.3(5.3,15.7) | 28115(15726,47295) | 340.8(190.7,573.8) | 722(379,1287) | 8.7(4.6,15.5) |
| Police conflict and executions | 159(89,275) | 2.1(1.1,3.6) | 4007(2258,6997) | 48.9(27.7,85.3) | 77(40,127) | 0.9(0.5,1.6) |
| Note: Adverse effects of medical treatment have no data in GBD. | | | | | | |
